# E3 Ubiquitin Ligases: Structures, Biological Functions, Diseases, and Therapy

**DOI:** 10.1002/mco2.70528

**Published:** 2025-12-07

**Authors:** Haochen Wang, Junbo Peng, Hongchan Li, Yuzhe Lan, Jing Guo, Qiang Qiu, Xuan Huang

**Affiliations:** ^1^ The MOE Basic Research and Innovation Center For the Targeted Therapeutics of Solid Tumors, Jiangxi Provincial Key Laboratory of Bioengineering Drugs, Institute of Translational Medicine, Jiangxi Medical College Nanchang University Nanchang China; ^2^ The Queen Mary School, Jiangxi Medical College Nanchang University Nanchang China; ^3^ Chongqing Research Institute Nanchang University Chongqing China; ^4^ Department of Dental General and Emergency, Jiangxi Province Key Laboratory of Oral Biomedicine, Jiangxi Province Clinical Research Center for Oral Diseases, The Affiliated Stomatological Hospital, Jiangxi Medical College Nanchang University Nanchang China; ^5^ Department of Hematology, State Key Laboratory of Biotherapy and Cancer Center, West China Hospital Sichuan University Chengdu China

**Keywords:** cancer, E3 ubiquitin ligase, neurodegenerative diseases, PROTAC, targeted protein degradation

## Abstract

E3 ubiquitin ligases are pivotal regulators within the ubiquitin–proteasome system, conferring specificity to protein ubiquitination and subsequent degradation, thereby maintaining cellular homeostasis. Their structural diversity allows for the precise control of vital processes, including the cell cycle, immune responses, and signal transduction, across various tissues. Despite their profound influence on physiology, a systematic understanding of how specific E3 ligases contribute to distinct disease pathogenesis and their translational potential remains incomplete. This review systematically delineates the classification and catalytic mechanisms of major E3 ligase families, including RING, HECT, and RBR types, and elaborates their pathological roles in driving carcinogenesis, cardiovascular remodeling, autoimmune dysregulation, metabolic syndrome, and neurodegenerative aggregation. We further synthesize recent advances in therapeutic modalities, from small‐molecule inhibitors targeting ligases like MDM2 to novel strategies in targeted protein degradation, notably proteolysis‐targeting chimeras (PROTACs) that hijack E3 machinery. By integrating mechanistic insights with emerging therapeutic landscapes, this work underscores the central role of E3 ligases in human diseases and provides a strategic framework for developing next‐generation, mechanism‐based therapeutics.

## Graphical abstract

E3 ligases function as critical regulators of cellular protein degradation, and their dysregulation is a core pathogenic mechanism in a wide array of human diseases, including cancer, cardiovascular conditions, neurodegenerative disorders, and autoimmune diseases. Due to their central role in pathology, E3 ligases have become highly compelling therapeutic targets. Consequently, novel interventions aimed at modulating their activity, particularly small‐molecule inhibitors and targeted protein degradation technologies such as PROTACs, are rapidly being developed, offering new promise for treating these major diseases.

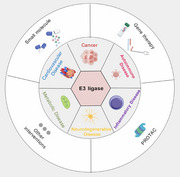

## 1 Introduction

Prior to the 1980s, intracellular protein degradation was widely regarded as a nonspecific, terminal process [1]. This perception was fundamentally transformed by the discovery of ubiquitin, which unveiled an entirely new field of regulated proteolysis and catalyzed an explosion of research throughout the 1990s [2]. The profound significance of this discovery was recognized with the 2004 Nobel Prize in Chemistry, awarded to Aaron Ciechanover, Avram Hershko, and Irwin Rose for their elucidation of ubiquitin‐mediated protein degradation [3]. Their work established the ubiquitin–proteasome system (UPS) as a major pathway for highly specific and selective protein degradation, central to maintaining cellular protein homeostasis [4]. Among these, E3 ubiquitin ligases are crucial for determining substrate specificity in the UPS, acting as the primary gatekeepers that decide which proteins are tagged for degradation [5]. More than 600 distinct E3 ligases have been identified in the human genome, belonging to structurally and mechanistically diverse families [6]. These ligases achieve specificity by recognizing defined sequence motifs or structural features within their target proteins, enabling them to selectively bridge ubiquitin‐charged E2 enzymes to appropriate substrates for ubiquitination [5, 7]. Through this role, E3 ligases serve as molecular switches that govern the stability and activity of numerous proteins, positioning them as central regulators of cellular physiology [8].

Given this pivotal regulatory function, it is not surprising that dysregulation of E3 ubiquitin ligases is increasingly implicated in the pathogenesis of human disease [9]. Mutations, altered expression, or disrupted control of E3 ligases can lead to the inappropriate stabilization of oncogenic proteins or excessive degradation of tumor suppressors, thereby driving malignant transformation [10]. Similarly, impaired activity of specific E3 ligases contributes to the accumulation of toxic protein aggregates in neurodegenerative disorders such as Alzheimer's and Parkinson's diseases (AD and PD) [11, 12]. Moreover, aberrant E3 ligase function is implicated in inflammatory and autoimmune conditions, cardiovascular diseases, and metabolic syndromes, underscoring their broad clinical relevance [13, 14, 15].

The pivotal role of E3 ubiquitin ligases in the pathogenesis of numerous diseases has positioned them at the forefront of therapeutic innovation [10]. Their enzymatic nature and intrinsic substrate specificity make them highly attractive targets for pharmacological intervention [10, 16]. Current therapeutic strategies are diverse, ranging from small‐molecule inhibitors that block catalytic activity or protein–protein interactions, to novel modalities like targeted protein degradation (TPD) [17, 18]. A landmark in TPD is the development of proteolysis‐targeting chimeras (PROTACs). These bifunctional molecules hijack the endogenous protein degradation machinery by simultaneously recruiting a target protein to an E3 ligase, leading to its ubiquitination and subsequent proteasomal destruction [19, 20].

In summary, given the critical functions of E3 ligases in human diseases and the rapid progress in developing therapies that exploit them, a comprehensive understanding of their biology and therapeutic potential is imperative. This review aims to synthesize the latest developments in this dynamic field. We begin by introducing the classification, structure, and fundamental biological functions of major E3 ligase families. Subsequently, we delve into the pathological consequences of E3 ligase dysregulation across a spectrum of major human diseases, including cancer, neurodegenerative, cardiovascular, autoimmune, and metabolic disorders. Building on this pathological foundation, we then discuss the expanding landscape of therapeutic strategies targeting E3 ligases, covering both traditional inhibitors and emerging TPD technologies like PROTACs. Finally, we summarize the current challenges and future directions in the field, with the aim of fostering deeper mechanistic insights and accelerating the translation of E3 ligase‐modulating therapies from bench to bedside.

## 2 The Classification and Structure of E3 Ligase

**FIGURE 1:**
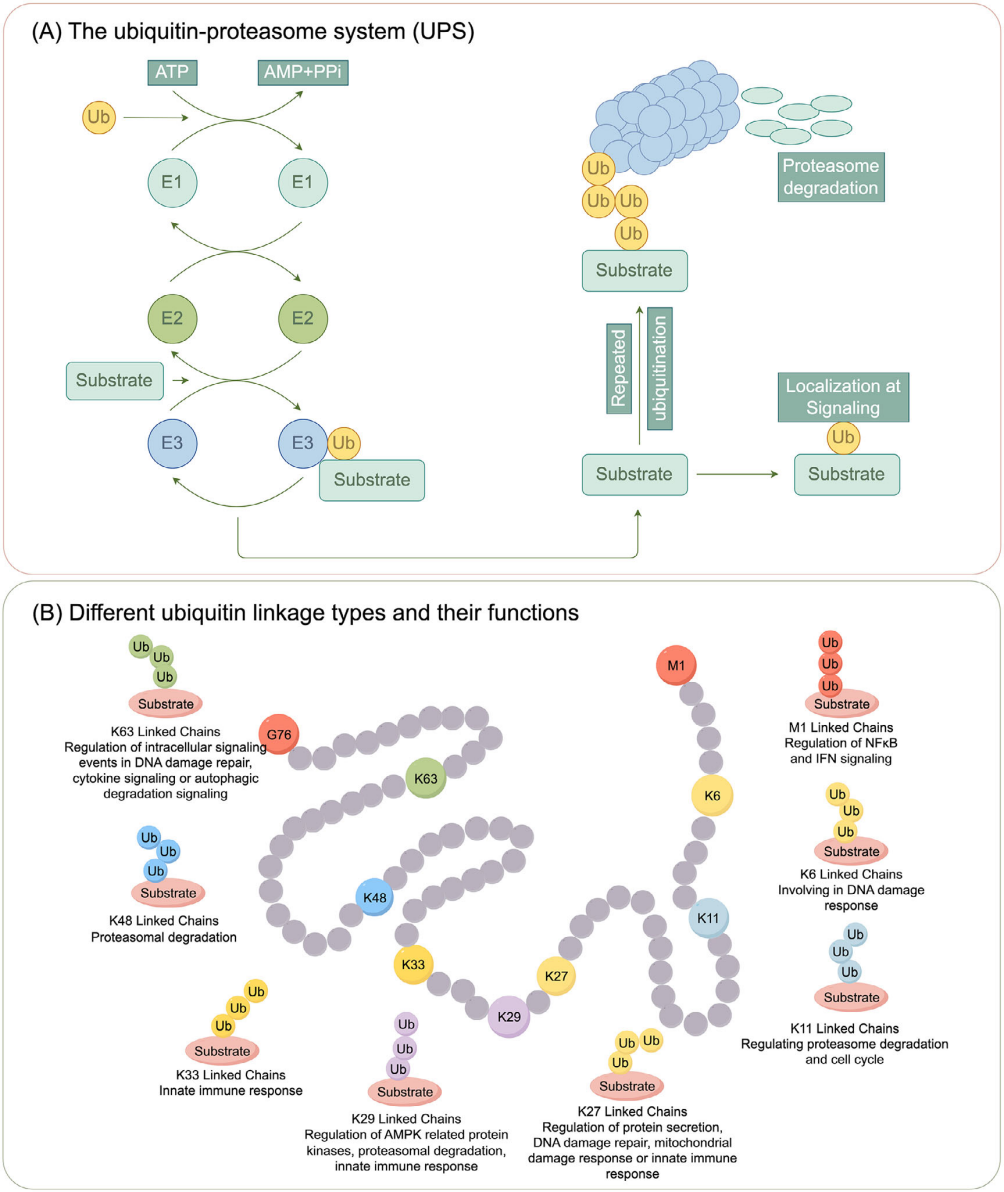
The role of E3 ubiquitin ligases in the ubiquitination pathway and types of ubiquitin chains. (A) Ubiquitin modification involves a sequential cascade of three enzymatic reactions performed by ubiquitin‐activating enzymes. The process begins when the ubiquitin‐activating enzyme (E1) transiently associates with ubiquitin, which is then transferred to the ubiquitin‐conjugating enzyme (E2). In collaboration with ubiquitin‐protein ligases (E3), E2 facilitates the conveyance of ubiquitin to specific target proteins via various mechanisms. Finally, the ubiquitin‐tagged proteins are then identified by proteasomes for degradation. (B) Ubiquitin (Ub) is a small, conserved protein consisting of 76 amino acids, common across eukaryotes. It contains seven lysine residues (K6, K11, K27, K29, K33, K48, K63) and an N‐terminal methionine (Met1), which serve as functional sites for various ubiquitin chain formations. These linkages enable ubiquitin to attach to target substrates through specific lysine sites, triggering distinct biological processes.

The UPS is a crucial posttranslational regulatory mechanism essential for maintaining cellular homeostasis and governing diverse biological processes such as cell differentiation, division, and adaptive immunity [21, 22, 23]. Dysregulation of the UPS is implicated in numerous human diseases, particularly cancer [24]. Ubiquitination, an ATP‐dependent process, unfolds through a cascade of transferring process, which includes three enzymatic reactions executed by ubiquitin‐activating enzyme (E1), ubiquitin‐conjugating enzyme (E2), and ubiquitin‐ligating enzymes (E3) [25, 26]. Ubiquitin, a 76 amino acid protein that is highly conserved, binds to target proteins and marks them for degradation through covalent post‐translational modification (PTM) [22, 25]. As ATP serves as the energy source, ubiquitin‐activating enzyme E1 triggers ubiquitin molecules and transfers them to the cysteine residue of ubiquitin‐conjugating enzyme E2. Ubiquitin ligase E3 then identifies the E2 complex and attaches the ubiquitin to lysine residues of the substrate proteins [9, 27]. This interaction enables the attachment of ubiquitin via a covalent bond, formed between the C‐terminal glycine residue of ubiquitin and the ε‐amino group of lysine on the substrate protein (Figure 1A) [28, 29]. E3 ligases catalyze the formation of polyubiquitin chain on substrate protein by attaching additional ubiquitin molecules to one of the seven lysine residues (K6, K11, K27, K29, K33, K48, K63) or the N‐terminal methionine (M1) of the preceding ubiquitin (Figure 1B) [30, 31]. These chains can be linear and branched, with distinct linkages specifying different biological outcomes. For instance, chains linked through K48 and K11 typically signal for protein degradation, while K63‐linked chains are involved in processes such as signal transduction, DNA repair, and immune responses [32, 33]. K11‐linked chains, in particular, are pivotal for regulating the cell cycle, ensuring the timely degradation of specific substrates that control the progression of mitosis and cytokinesis [34, 35]. K48‐linked chains are primarily involved in tagging proteins for proteasomal degradation, thereby maintaining cellular protein homeostasis by removing damaged, misfolded, or unneeded proteins [36, 37]. In contrast, K63‐linked chains have been extensively linked to signaling pathways, including the regulation of DNA damage response, inflammation, and cellular stress responses, highlighting their nondegradative role [38]. Additionally, K6, K27, and K33 linkages have emerged as regulators of protein interactions and cellular responses to external stimuli [39, 40, 41]. The formation and function of these ubiquitin chains depend on the activity of specific E3 ligases and can be regulated by deubiquitinating enzymes (DUBs), which alter these processes [42]. By controlling the length and type of ubiquitin chains, DUBs regulate a variety of cellular processes, including the stability, localization, and activity of many proteins [43]. Finally, 26S proteasomes identify and target the labeled substrates for degradation. This process is primarily driven by various types of ubiquitin linkages, including K11, K29, and K48 polyubiquitin chains, with K48 type being the most well studied and efficient for bulk degradation [44]. Additionally, substrates with unstructured or loosely folded segments are preferentially engaged by the proteasome, which helps facilitate their translocation and degradation.

Among UPS components, E3 ligases are of particular significance, as they are responsible for the specific identification of substrates, determining which proteins are selected for ubiquitination [45]. Beyond their canonical role is in protein turnover, E3 ligases regulate diverse processes such as cell cycle progression, DNA damage response, signal transduction, and immune surveillance [46, 47]. For instance, the regulation of the G1‐S checkpoint by E3 ligases ensures the timely progression of the cell cycle, while their involvement in the DNA damage response pathways facilitates the repair or elimination of damaged cells to maintain genomic integrity. Additionally, E3 ligases are key players in cellular signaling, modulating the activities of receptors, kinases, and transcription factors in response to extracellular stimuli. This enables the selective degradation of damaged or misfolded proteins, translating cellular signals into outcomes that maintain protein homeostasis [48]. Consequently, their dysregulation is linked to oncogenesis, neurodegenerative diseases, and immune dysfunctions, highlighting their central role in cellular homeostasis and disease pathogenesis [49, 50, 51]. Furthermore, defects in E3 ligase‐mediated regulation of immune responses can result in immune system dysfunction, contributing to autoimmune diseases or impaired antitumor immunity [15, 52].

**FIGURE 2:**
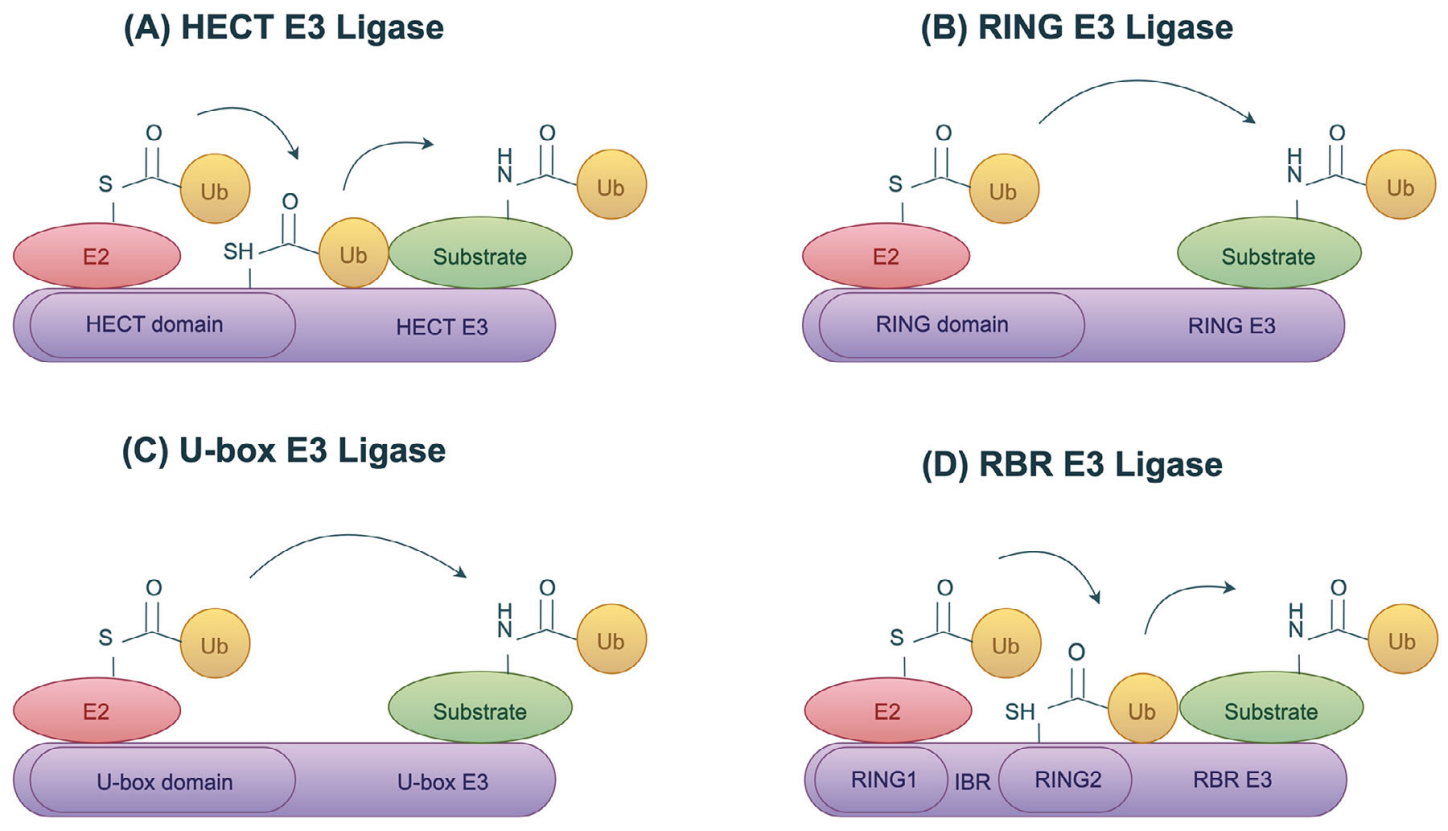
The classification of E3 ligases. E3 ligases are classified into four main isoforms based on their structural and biochemical characteristics: HECT type (A), RING finger type (B), U‐box type (C), and RBR type (D).

Over six hundred E3 ubiquitin ligases have been discovered in the human proteome, which can be categorized into four main distinct categories: homologous to E6‐associated protein C‐terminus (HECT) type, really interesting new gene (RING) finger type, U‐box type, RING‐in‐between‐RING (RBR) type (Figure 2) [9]. In addition to these main types, there are some other E3 ubiquitin ligases, such as plant homeodomain (PHD) containing ligases [53]. Each type of E3 ligases recognizes substrates and regulates their degradation through different mechanisms and structural domains [54, 55, 56]. RING E3 ligases are the major type of E3 ligases, characterized by their RING domain [57]. During the ubiquitination process, the RING domain of the RING E3 ligase binds to the E2 conjugase enzyme, allowing ubiquitin to bypass the formation of an E3–Ub intermediate and be transferred directly from E2 onto the target substrate [7, 58]. U‐box E3 ligase is a variant of the RING type that shares a similar structure but lacks the Zn^2^⁺ ion found in the RING domain [59]. The U‐box E3 ligase serves as an interaction site with E2 during the catalyzed ubiquitination process. Once this interaction occurs, ubiquitin is directly transferred from E2 to a specific lysine residue on the substrate protein [60]. HECT E3 ligases exert their function through their conserved C‐terminal HECT domain, which contains an active cysteine site. Activated E2 can transfer ubiquitin to an active cysteine site on the HECT domain before binding to the target substrate [61, 62]. Subsequently, the E3 ligase transfers this ubiquitin to the target substrate, which is recognized and bound by its N‐terminal domain [63]. RBR E3 ligases combine features of both RING and HECT types, containing the RING1, IBR, and RING2 domains, and transfer ubiquitin to the substrate in a manner similar to HECT after binding to E2 [64, 65]. The RING1 domain is responsible for recruiting ubiquitin‐conjugating E2 enzymes, and the RING2 domain contains a crucial catalytic cysteine. The IBR domain has a similar structural fold to RING2, but it lacks the catalytic cysteine residue. The main differences between these types of E3 ligases lie in their catalytic mechanisms and structural characteristics.

## 3 The Role of E3 Ligases in Carcinogenesis

**FIGURE 3:**
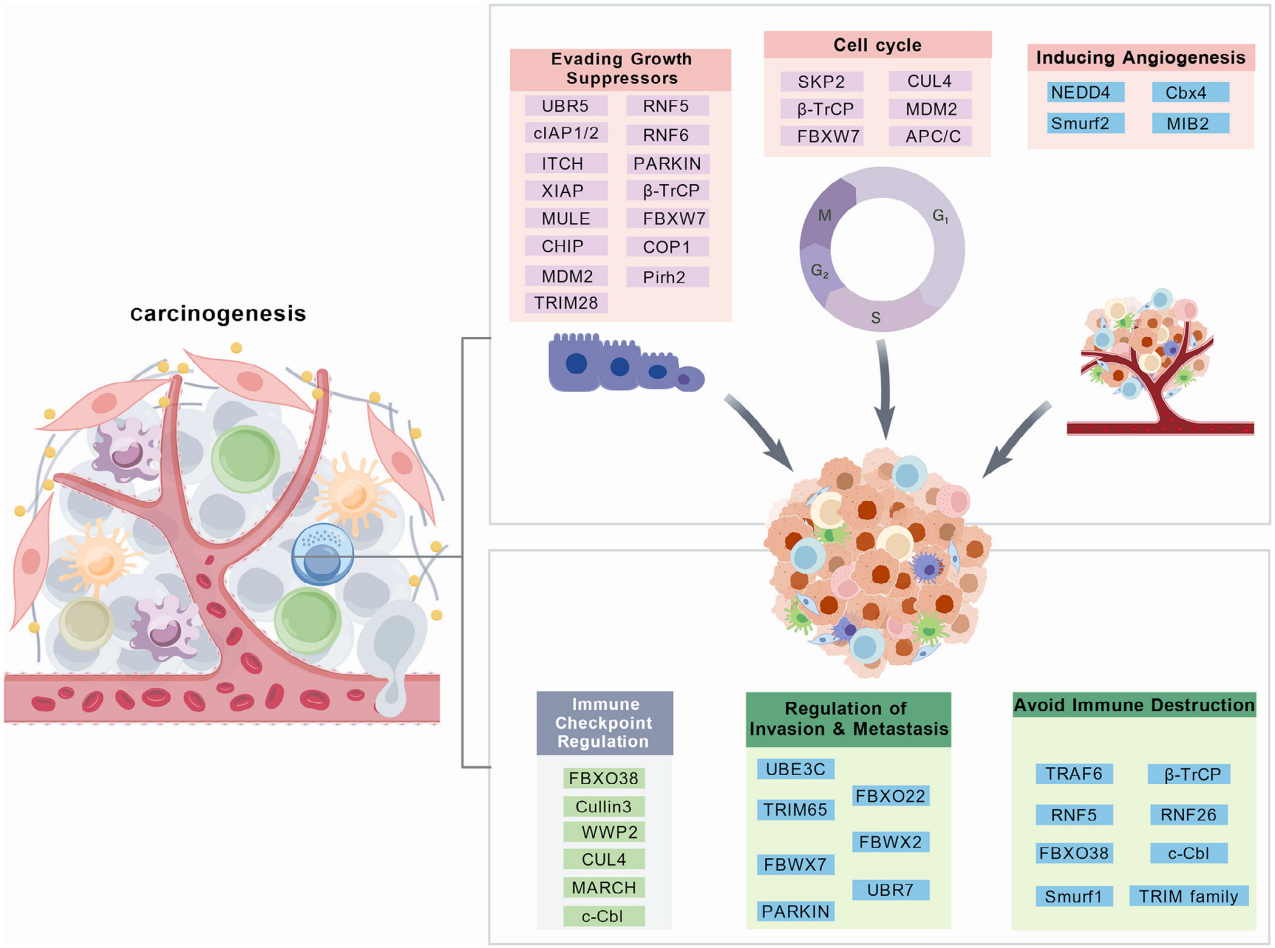
The multifaceted roles of E3 ubiquitin ligases in the hallmarks of cancer. The multifaceted roles of E3 ubiquitin ligases in carcinogenesis. The figure illustrates the diverse and critical functions of E3 ubiquitin ligases in modulating the hallmarks of cancer. These enzymes act as central regulators of key oncogenic processes, including dysregulation of the cell cycle (e.g., APC/C, SKP2), evading growth suppressors (e.g., MDM2, FBXW7), inducing angiogenesis (e.g., NEDD4, Smurf2), and activating invasion and metastasis (e.g., UBE3C, PARKIN). Furthermore, E3 ligases are crucial for tumor–immune interactions, participating in both the regulation of immune checkpoints (e.g., c‐Cbl, FBXO38) and the broader mechanisms for avoiding immune destruction (e.g., TRAF6, TRIM family). Each panel lists representative E3 ligases that drive or suppress these processes, underscoring their position as pivotal hubs in cancer progression and as potential therapeutic targets.

Cancer is a group of diseases characterized by the uncontrolled growth and spread of abnormal cells that can invade nearby tissues and potentially spread to other parts of the body [66]. To sustain this malignancy, cancer cells operate under immense proteotoxic stress, leading to a higher protein turnover rate and a critical dependency on the UPS for survival [67]. The critical reliance of cancer cells on the UPS for survival establishes it as a major vulnerability and a validated therapeutic target, whose potential for specificity is conferred directly by the E3 ubiquitin ligases [68, 69]. Consequently, the dysregulation of this E3‐mediated proteolytic system is a well‐established driver of oncogenic transformation [70]. E3 ligases exhibit a functional dichotomy in cancer, acting as either oncogenes or tumor suppressors [70]. An E3 ligase assumes an oncogenic role when it mediates the degradation of proteins that restrain cell proliferation or promote cell death, such as tumor suppressors [68, 70]. A prime example is the overproduction of the E3 ligase Murine Double Minute 2 (MDM2), which leads to the destruction of the p53 tumor suppressor and dismantles a key barrier against cancer development [71]. Conversely, an E3 ligase functions as a tumor suppressor when its substrates are oncoproteins. In this capacity, the ligase eliminates key drivers of malignant progression. The loss of function of such an E3 ligase, for instance, the E3 ligase anaphase‐promoting complex/cyclosome (APC/C), which targets cyclins to enforce cell cycle checkpoints, leads to the aberrant accumulation of its oncogenic substrates, fueling uncontrolled cell division [72]. E3 ligases are critical cancer drivers that regulate hallmark cancer processes, though their specific functions depend on the cellular environment and substrate condition. Below, we highlight the role of E3 ligases in several of these critical hallmarks (Figure 3).

### 3.1 Cell Cycle Progression

E3 ubiquitin ligases play a central role in regulating the cell cycle by conferring substrate specificity within the UPS, thereby ensuring the unidirectional progression through the G1, S, G2, and M phases [73]. The cyclin–CDK oscillator is regulated by a range of E3 enzymes that recognize phospho‐regulated degrons or defined destruction motifs in key regulators and target them for proteasomal degradation, ensuring irreversible transitions between cell‐cycle stages [74, 75].

During the process of mitotic exit and the G1 phase, the APC/C acts in concert with its coactivators CDC20 and cadherin (CDH)1 [76]. APC/C^CDC20^ is first activated in early mitosis upon CDK1‐mediated phosphorylation of APC/C subunits, thereby promoting the K11‐linked polyubiquitination and subsequent degradation of cyclin A, cyclin B, and securin to trigger anaphase onset [77, 78]. Securin degradation liberates separase, which in turn cleaves the cohesin subunit Scc1 to trigger sister chromatid separation. Subsequently, the destruction of cyclin B results in the elimination of mitotic CDK activity, thus activating APC/C^CDH1^ to destroy key mitotic regulators, such as Cdc20 [79, 80]. As CDK1 activity declines, CDC14 phosphatase‐driven dephosphorylation unmasks CDH1, allowing APC/C^CDH1^ to target the remaining mitotic kinases (PLK1, Aurora A/B) and coactivator CDC20 itself for destruction, thus enforcing mitotic exit and resetting CDK activity to its low G1 state [81]. In late G1, the accumulation of the early mitotic inhibitor EMI1 temporarily restrains APC/C^CDH1^ until EMI1 is phosphorylated and ubiquitinated by SCF^β‑TrCP^, relieving APC/C inhibition and permitting S‑phase cyclin A synthesis [82, 83]. Subsequently, the SCF (SKP1–CUL1–F‐box) complexes become the primary regulators of G1/S transition.

In late G1, CDK2 phosphorylates SKP2 to shield it from APC/C^CDH1^‐mediated turnover, thereby activating the SCF^SKP2^ complex [84]. Once engaged, the SCF^SKP2^ complex drives the G1/S transition by ubiquitinating and degrading the Cip/Kip inhibitors p21^CIP1^, p27^KIP1^, and p57^KIP2^, freeing CDK2–cyclin E/A to initiate DNA synthesis [85, 86, 87]. Throughout G1, both SCF^SKP2^ and its counterpart SCF^FBXW7^ regulate cyclin E levels via proteasomal degradation, ensuring precise control over S‐phase entry [88, 89]. Additionally, these E3 ligases exert opposite effects on c‐MYC, SCF^SKP2^ enhances c‐MYC stability and transcriptional output, whereas SCF^FBXW7^ targets c‐MYC for proteasomal destruction [90, 91].

Other cullin‐based E3 ligases are also involved in the cell‐cycle control. CUL3–RhoBTB3 complexes contribute to cyclin E turnover, CUL4–DCAF11 and CUL4–DDB2 ligases degrade p21 and p27, respectively, fine‐tuning S‑phase entry, while MDM2, a RING‐type E3, targets the p53 tumor suppressor to modulate G1 checkpoint integrity [92, 93, 94, 95, 96]. These E3 ligases form interconnected feedback loops. Cyclin–CDK activity promotes EMI1 turnover via SCF^β‑TrCP^, which in turn activates APC/C to degrade mitotic cyclins and its own activators, while APC/C^CDH1^ cooperates with SCF to restrain CDKs in the ensuing G1 and primes cells for another round of division [83, 97, 98, 99].

Mechanistically, APC/C and SCF complexes share a RING‐finger subunit (APC11 or ROC1/RBX1) that recruits E2‐Ub conjugates and a cullin scaffold (APC2 or CUL1‐5) that organizes the catalytic core and connects to substrate adaptors [100, 101]. In SCF, SKP1 bridges cullin to various F‑box proteins that recognize phosphorylated degrons, whereas APC/C employs WD40‐repeat coactivators CDC20/CDH1 to engage D‐box and KEN‐box motifs, their association tightly controlled by phosphorylation and mitotic checkpoint signals [102, 103]. By eliminating previous cell‐cycle drivers and converting kinase signals into irreversible proteolysis, E3 ligases ensure the accuracy and timing of chromosome segregation, DNA replication, and cell division.

### 3.2 Evasion of Growth Suppressors

Evading growth suppressors is a fundamental hallmark of cancer, allowing tumor cells to bypass the normal cellular mechanisms that limit proliferation [104, 105]. Several E3 ligases act as tumor suppressors by negatively regulating cell growth and are often inactivated in human malignancies [106]. The two prototypical tumor suppressors, RB transcriptional corepressor1 (Rb) and TP53, serve as central nodes in regulatory circuits that govern the fundamental cell fate decision between proliferation and the activation of senescence or apoptosis [104]. Consequently, the inactivation of tumour suppressor genes removes these cellular checkpoints, a critical step that promotes tumourigenesis by eliminating essential mechanisms that inhibit cell proliferation [107].

The tumor suppressor protein p53 is a key regulator of signaling pathways, essential for maintaining cellular homeostasis, and its functional inactivation is found in most human cancers [108]. The activity and stability of p53 are primarily controlled by UPS through PTMs [109]. The archetypal antagonist of p53 is the RING‐finger E3 ubiquitin ligase MDM2, which functions as the main negative regulator by maintaining low basal levels of p53 in unstressed cells [110]. Mechanistically, MDM2 possesses a p53‐binding domain at its N‐terminus and a C‐terminal RING domain with E3 ubiquitin ligase activity. The N‐terminal domain of MDM2 binds to p53, thereby inhibiting its transcriptional activity [111]. Concurrently, the C‐terminal RING domain, with its intrinsic E3 ubiquitin ligase functionality, recruits E2 ubiquitin‐conjugating enzymes, which in turn facilitate the ubiquitination and subsequent proteasomal degradation of p53 [112]. Moreover, low MDM2 levels catalyze p53 monoubiquitination, triggering its nuclear export, whereas the high concentrations often found in tumors with MDM2 amplification led to polyubiquitination and direct proteasomal degradation [112, 113]. This interaction forms a critical autoregulatory feedback loop, as MDM2 is a transcriptional target of p53. Furthermore, MDM2 activity is frequently potentiated by its homolog, MDM4 (or MDMX), which oligomerizes with MDM2 to form a more potent E3 ligase complex for p53 degradation [114, 115]. This MDM2/MDM4 axis, which is often hyperactive in cancers like soft‐tissue sarcomas, glioblastomas, and breast cancer, has become a major target for therapeutic intervention, with strategies focused on nongenotoxic p53 reactivation [116, 117]. Consequently, the pharmacological disruption of the MDM2–p53 interaction is a key therapeutic strategy in the treatment of cancer. Small‐molecule inhibitors, such as the nutlin family and AMG‐232, have been developed to competitively occupy the p53‐binding pocket of MDM2, thereby preventing p53 degradation [118].

In addition to MDM2, a series of E3 ligases participate in the regulation of p53. E3 ligases such as COP1 and Pirh2 can ubiquitinate p53 independently and can also function synergistically with MDM2 to enhance its suppression [119, 120]. Moreover, other E3 ligases act as crucial cofactors; for instance, TRIM28 binds MDM2 to promote p53 ubiquitination and degradation, thereby facilitating tumor proliferation [121]. The regulation of oncogenic mutant p53 proteins is another important function of E3 ligases. The cochaperone E3 ligase CHIP, a U‐box type ligase, cooperates with the Hsp70 chaperone to mediate the degradation of misfolded p53 mutants [122, 123]. This activity is opposed by the Hsp90 chaperone, which shields mutant p53 from CHIP‐mediated degradation [123]. These dynamic positions the chaperone machinery as a therapeutic vulnerability, inhibiting Hsp90, for instance, can unleash CHIP's E3 ligase activity, promoting the selective degradation of mutant p53 and highlighting a distinct therapeutic strategy for tumors harboring p53 mutations [124].

Rb is a key tumor suppressor and a primary negative regulator of cell cycle, differentiation, and apoptosis [125, 126, 127]. Although the inactivation of Rb through phosphorylation has been well established, recent evidence reveals a parallel and crucial mechanism governed by E3 ubiquitin ligases that directly mediate Rb's proteolytic degradation. Two key E3 ligases, UBR5 and NRBE3, have been identified as central players in this process. UBR5 contributes to the progressive decrease in Rb concentration during early G1, a necessary step that lowers the threshold for G1‐S transition and sensitizes cells to CDK4/6 activity [128, 129]. Concurrently, the novel Rb E3 ubiquitin ligase NRBE3 directly targets Rb for destruction via the ubiquitin–proteasome pathway [129]. NRBE3 utilizes a canonical LXCXE motif for Rb binding and possesses an essential U‐box for its E3 ligase activity [129]. This interaction is part of a feed‐forward loop, as NRBE3 is transcriptionally activated by E2F1, the very transcription factor released upon Rb inactivation, thereby accelerating the G1/S transition [129].

The regulation of Rb also involves other PTMs and E3 ligases, particularly in cancer, where its pathway is frequently impaired. E3 ligase TRIM28 has been shown to specifically target phosphorylated Rb (p‐Rb) to promote its ubiquitination and subsequent degradation [130]. This process is counter regulated by SETDB1, which protects p‐Rb from destruction, thereby creating a dynamic balance particularly notable in prostate cancer [131]. This interaction reveals a potential therapeutic strategy that combining inhibition of SETDB1 and CDK4/6 could synergistically suppress tumour growth by preventing Rb degradation [132]. E3 ligases also indirectly control Rb, RNF6 promotes the degradation of the CDK inhibitor p27 [133]. The removal of p27 unleashes CDK2/Cyclin E activity, leading to increased Rb phosphorylation and inactivation [133, 134]. Therefore, these findings demonstrate that E3 ubiquitin ligases regulate the Rb tumor suppressor axis through a complex network of direct degradation and indirect regulation of its phosphorylation state.

The survival of a cell depends on the balance between prosurvival members and proapoptotic members. E3 ligases have been demonstrated to play a pivotal role in regulating cellular survival. Mcl‐1 is an essential prosurvival E3 ligase protein [135]. Its transient nature is critical for permitting apoptosis, and accordingly, it is targeted by a multitude of E3 ligases, including MULE (ARF–BP1), Parkin, and the SCF^β‐TrCP^ and SCF^FBW7^ complexes [136, 137]. This multifaceted regulation ensures that Mcl‐1 levels can be rapidly diminished to sensitize cells to death signals [138]. Similarly, the prosurvival protein Bcl‐2 is itself targeted for degradation by the E3 ligase XIAP, a key interaction that links the Bcl‐2 family directly to the inhibitor of apoptosis (IAP) protein machinery [139, 140].

Conversely, E3 ligases can restrain apoptosis by targeting proapoptotic Bcl‐2 family members. For instance, the HECT E3 ligase ITCH specifically recognizes and ubiquitinates tBID, thereby preventing the amplification of death receptor signals to the mitochondria [141]. Furthermore, E3 ligases such as Parkin and IBRDC2 have been shown to target the effector protein BAX [142]. By promoting its degradation, these ligases limit the pool of BAX available for mitochondrial translocation and activation, effectively raising the threshold required to trigger mitochondrial outer membrane permeabilization and initiate apoptosis [143, 144].

The final executioners of apoptosis are caspases, a family of proteases that are held in check by the IAP proteins [145]. Several IAPs, including the well‐characterized XIAP and cIAP1/2, possess a C‐terminal RING domain, granting them E3 ligase activity [146, 147]. Therefore, they have a dual function that they directly bind and inhibit caspases while also functioning as E3 ligases to mediate protein degradation.

As E3 ligases, IAPs can target active caspases for ubiquitination and degradation, providing a robust mechanism to extinguish low‐level, unwanted caspase activity [148, 149]. Furthermore, they create a suppressive cellular environment by targeting their own natural antagonists. For instance, upon release from the mitochondria during apoptosis, the IAP antagonist Smac/DIABLO binds to and neutralizes IAPs. However, this binding can also trigger the cIAP1/2‐mediated ubiquitination and degradation of Smac itself, forming a negative feedback loop [150, 151]. Similarly, XIAP targets its potent antagonist, ARTS, for proteasomal clearance to maintain cell viability [152, 153].

The IAPs themselves are also regulated by ubiquitination. The tumor suppressor ARTS can act as an adaptor, recruiting the E3 ligase SIAH to XIAP, leading to auto‐ubiquitination and degradation of XIAP [154, 155]. This mechanism enables proapoptotic signals (stabilized ARTS) to degrade their own inhibitory factors, thereby releasing caspases to execute the death program [156].

### 3.3 Regulation of Invasion and Metastasis

The metastatic cascade represents a complex, multistep process that relies on the dynamic regulation of protein networks controlling cell adhesion, migration, and survival [157]. E3 ligases, by conferring substrate specificity to the UPS, serve as critical regulators of this process. Their functions exhibit duality, serving both as potent inhibitory factors and as drivers of metastasis, with this distinction determined by their substrates and cellular environment.

#### 3.3.1 E3 Ligases as Barriers to Metastasis

A key class of metastasis‐suppressing E3 ligases functions by targeting and dismantling core oncogenic and prometastatic signaling pathways. Members of the SCF complex are particularly prominent in this role, FBW7 promotes the degradation of a broad spectrum of oncoproteins [158]. Its tumor‐suppressive capacity extends to curtailing the epithelial–mesenchymal transition (EMT) by targeting the chromatin remodeler Brg1 for proteolysis, thereby preventing the transcriptional activation of the EMT master regulator Snail [159, 160]. Furthermore, FBW7 can influence posttranscriptional gene regulation by degrading the N6‐methyladenosine (m6A) reader YTHDF2, thereby impeding m6A‐mediated mRNA decay and suppressing cancer cell propagation [161]. Similarly, the F‐box protein FBXW2 restrains invasion by targeting key signaling molecules like β‐catenin and the kinase TAK1 for degradation [162]. Notably, these suppressive E3 ligases are often embedded in complex regulatory circuits. FBXW2 itself is a substrate for the oncogenic E3 ligase β‐TrCP1, establishing a hierarchical degradation cascade that fine‐tunes the balance between tumor suppression and progression [163].

Beyond the direct degradation of oncoproteins, suppressive E3 ligases also modulate cellular metabolism and epigenetic landscapes [164, 165]. Parkin, an E3 ligase implicated in PD, exhibits tumor suppressor functions by suppressing metabolic reprogramming [165]. It directly targets and degrades key metabolic enzymes, including phosphoglycerate dehydrogenase and pyruvate kinase M2 (PKM2), to inhibit aberrant serine synthesis and glycolysis, respectively [166]. Parkin also counteracts the hypoxic response, essential for tumor survival and angiogenesis, by mediating the degradation of hypoxia‐inducible factor 1α (HIF‐1α) [167]. In a distinct mechanism operating at the chromatin level, UBR7 suppresses EMT by catalyzing the monoubiquitination of histone H2B at lysine 120 (H2BK120Ub) [168, 169]. This epigenetic mark promotes the transcription of the cell‐adhesion molecule CDH4, which not only strengthens cell–cell junctions but also sequesters β‐catenin in the cytoplasm, effectively dampening prometastatic Wnt signaling [170, 171].

#### 3.3.2 E3 Ligases as Drivers of Metastasis

A series of E3 ligases act as oncogenes, actively promoting metastasis. These enzymes typically exert their effects by disrupting tumor suppression mechanisms or activating invasion‐promoting pathways. The HECT domain E3 ligase UBE3C, for example, enhances cancer stemness and metastasis by targeting the p53 cofactor AHNAK for degradation, thereby relieving transcriptional repression of stemness‐related genes [172, 173]. UBE3C also fuels Wnt signaling by degrading the destruction complex component AXIN1 [174].

Oncogenic E3 ligases also facilitate cell migration by remodeling the cellular architecture. By targeting the Rho GTPase‐activating protein ARHGAP35 for degradation, TRIM65 increases RhoA activity, leading to profound cytoskeletal reorganization and enhanced migratory capacity [175, 176, 177]. Moreover, F‐box protein FBXO22 is a prometastatic E3 ligase with multiple substrates [178]. It promotes tumorigenesis through several parallel mechanisms: degrading the nuclear pool of the tumor suppressor PTEN, eliminating the cell cycle inhibitor p21, and promoting angiogenesis by stabilizing HIF‐1α [179, 180]. In addition, FBXO22 can also deploy nondegradative ubiquitination to drive metastasis by mediating K63‐linked polyubiquitination of the kinase LKB1, a modification that inhibits the tumor‐suppressive LKB1–AMPK signaling axis [181]. E3 ligases utilize a variety of cellular processes, including cell cycle control, signal transduction, and angiogenesis, to create a cellular environment conducive to invasion and metastasis.

### 3.4 The E3 Ligase in Immune Checkpoint Regulation

Although immune checkpoint blockade (ICB) therapy has achieved significant success, primary and acquired resistance remains a major clinical challenge [182]. A central mechanism governing the efficacy of ICB is the surface expression of checkpoint proteins themselves, a process intricately controlled by PTMs [182, 183]. Among these, ubiquitination orchestrated by E3 ubiquitin ligases has emerged as a critical regulatory node [184]. E3 ligases determine the fate of checkpoint proteins by tagging them for proteasomal degradation, thereby directly modulating the immunogenicity of the tumor microenvironment and the activity of antitumor T cells [185, 186, 187].

The PD‐1/PD‐L1 axis serves as a paradigm of this regulatory process. The abundance of the PD‐1 receptor on activated T cells is actively curtailed by specific E3 ligases [188]. The F‐box protein FBXO38, a component of the SCF E3 ligase complex, and the Cbl‐family ligase c‐Cbl both mediate K48‐linked polyubiquitination of the PD‐1 cytoplasmic tail, targeting it for degradation and thus unleashing T cell activity [185, 189, 190]. Similarly, the expression of its ligand, PD‐L1, on tumor and antigen‐presenting (AP) cells is under tight ubiquitin‐dependent control [191]. The stability of PD‐L1 is famously coupled to its glycosylation status; unglycosylated PD‐L1 is phosphorylated by GSK3β, creating a phosphodegron recognized by the β‐TrCP E3 ligase for ubiquitination and degradation [187, 192]. Furthermore, other E3 ligases, including Cullin3^SPOP^ and members of the Cbl family, also contribute to PD‐L1 turnover, presenting multiple targets for therapeutic intervention [193].

Beyond the PD‐1/PD‐L1 axis, E3 ligases regulate various other critical immune checkpoints. The surface expression of CTLA‐4's ligands, CD80 and CD86, on AP cells is constrained by several membrane‐associated RING‐CH (MARCH) family E3 ligases, including MARCH1 and MARCH8, as well as the viral‐encoded ligase MIR2, which promote their endocytosis and lysosomal destruction [194, 195]. Likewise, the expression of major histocompatibility complex (MHC) molecules, which serve as ligands for both T‐cell receptors (TCRs) and checkpoint receptors, is exquisitely regulated by ubiquitination [196]. MHC‐II levels are suppressed by MARCH1, MARCH8, and the TMEM127/WWP2 complex, while MHC‐I is targeted for degradation by MARCH4, MARCH9, and the US2/TRC8 complex [197, 198]. CD47 is also subject to this regulation, with the DDB1–CUL4A complex targeting it for proteasomal destruction to enhance macrophage‐mediated phagocytosis [199]. In contrast, the specific E3 ligases that directly govern the stability of several key checkpoints, such as TIM‐3, remain a subject for future investigation [199]. Targeting E3 ligases that degrade checkpoint proteins or their ligands may serve as a complementary strategy for cancer treatment, offering potential to overcome drug resistance and broaden the efficacy of cancer immunotherapy.

### 3.5 E3 Ligases as Master Regulators of Immune Signaling Pathways

E3 ligases are essential components of immunomodulatory signaling pathways, converting extracellular stimuli into precise transcriptional and cellular responses. By controlling the activation, duration, and termination of signaling cascades such as NF‐κB, JAK–STAT, these E3 ligases are involved in inflammation‐induced carcinogenesis [199]. Dysregulation of these E3 ligases is a common feature in cancer, where they can be hijacked to promote chronic inflammation, immune evasion, and tumor progression.

The NF‐κB pathway, a master regulator of inflammation and immunity, is fundamentally dependent on ubiquitination for its activation. In the canonical pathway, signaling through receptors like the TCR or Toll‐like receptor (TLR)s converges on the activation of E3 ligases such as TRAF6, leading to the K63‐linked ubiquitination of upstream kinases and the IKK complex component NF‐κB essential modulator (NEMO) [200, 201, 202]. This process triggers the phosphorylation and subsequent degradative K48‐linked ubiquitination of the inhibitor IκB by the SCF‐β–TrCP E3 ligase complex, liberating NF‐κB dimers to enter the nucleus [202]. The noncanonical pathway is similarly controlled, where the stability of the central kinase NIK is governed by a complex of TRAF2, TRAF3, and cIAPs, which constitutively ubiquitinate NIK for degradation, thus leading to noncanonical NF‐κB activation [203, 204]. Furthermore, a number of E3 ligases within the TRIM family act as positive or negative regulators of the NF‐κB pathway, thereby modulating the pathway.

The JAK–STAT pathway, crucial for cytokine signaling, is also intricately modulated by E3 ligases, which often act as key negative feedback regulators [205]. The suppressor of cytokine signaling (SOCS) proteins possess E3 ligase activity and target both JAK kinases and cytokine receptors for proteasomal degradation [206]. Inhibiting SOCS1 gene expression in dendritic cells improves their antigen presentation and antitumor immune function [207]. Similarly, the protein inhibitors of activated STAT (PIAS) family can function as E3 SUMO ligases to inhibit STAT1 activity [208]. While the phosphorylation of STAT1 is associated with the polarization of M1 macrophages that suppress cancer, it can paradoxically facilitate tumor growth by mediating immune suppression, highlighting its dual regulatory effect on tumor activity [209, 210, 211]. Its activity can be suppressed through degradation mediated by ligases like Smurf1, or enhanced via nondegradative K63‐linked ubiquitination by ligases such as NGLAM [212, 213]. This highlights the fact that distinct ubiquitin modifications by different E3 ligases can lead to profoundly different functional outcomes.

E3 ligases also exert precise control over the innate immune STING pathway [214]. Activating K63‐linked ubiquitination of STING by TRIM56 and TRIM32 promotes downstream signaling and interferon (IFN) production [215, 216]. Conversely, degradative K48‐linked ubiquitination by RNF5 terminates the signal [217]. Furthermore, RNF26 applies a protective K11‐linked ubiquitin chain that shields STING from RNF5‐mediated degradation [218]. TRIM29, identified as the E3 ligase of STING, inhibits the production of IFN‐I by degrading STING, promoting the persistent infection of EBV in airway epithelial/dendritic cells [219]. TRIM29 deficiency enhances the antiviral response mediated by STING signaling and improves the resistance of mice to HSV‐1 infection [220]. In addition, TRIM30α mediates the K48 ubiquitination and degradation of STING, negatively regulating the anti‐DNA virus signaling pathway. TRIM30α‐deficient mice are also more resistant to HSV‐1 infection [221]. This complex regulation, which involves multiple E3 ligases and distinct ubiquitin linkages, highlights the importance of precisely regulating STING responses.

### 3.6 Inducing Angiogenesis

Tumor angiogenesis is the process by which tumors stimulate the growth of new blood vessels to supply them with oxygen and nutrients, allowing them to grow and spread [222]. This process is a hallmark of cancer and is critical for tumors to grow from a small size to metastasize to other parts of the body [222]. E3 ubiquitin ligases play crucial roles in regulating angiogenesis. The E3 ligase MIB2, in complex with the protocadherin FAT1, promotes the degradation of YAP/TAZ proteins in endothelial cells, thereby limiting excessive angiogenesis during development and tumor growth [223]. The loss of either FAT1 or MIB2 leads to increased YAP/TAZ activity and heightened angiogenic signaling [223]. NEDD4 in the heart is another E3 ligase that promotes angiogenesis and wound healing by ubiquitinating and degrading TSP1, a negative regulator of VEGF, whereas its deficiency, common in diabetic conditions, impairs these processes [224]. In mesenchymal stem cells from patients with ankylosing spondylitis, elevated E3 ligase Smurf2 enhances angiogenesis by degrading PTX3, a natural angiogenesis inhibitor [225]. Furthermore, as part of a cullin3‐based E3 ligase complex, BAZF mediates crosstalk between VEGF and Notch signaling pathways to support angiogenesis by promoting degradation of Notch pathway components [226]. The SUMO E3 ligase Cbx4 also enhances tumor angiogenesis by enhancing HIF‐1α activity and VEGF expression in hepatocellular carcinoma [227]. Collectively, these findings highlight the diverse and critical roles of E3 ligases in finely regulating angiogenic pathways, making them potential therapeutic targets for diseases involving abnormal blood vessels.

## 4 E3 Ligases in Cardiovascular Disease Pathogenesis

In the cardiovascular system, E3 ligases regulate key processes such as inflammation, cell death, hypertrophy, fibrosis, and immune responses, all of which are implicated in the pathogenesis of cardiovascular diseases [228, 229]. Because they specifically recognize and target substrate proteins for degradation, E3 ligases are crucial for maintaining cellular protein homeostasis, which is essential for normal cardiovascular function [230]. Dysregulation of E3 ligase activity disrupts this balance, leading to the accumulation of pathogenic proteins or the loss of protective proteins. This disruption is a key factor in the development and progression of multiple cardiovascular diseases, making E3 ligases an important area of ​​research for potential therapeutic intervention.

### 4.1 Heart Failure

**FIGURE 4:**
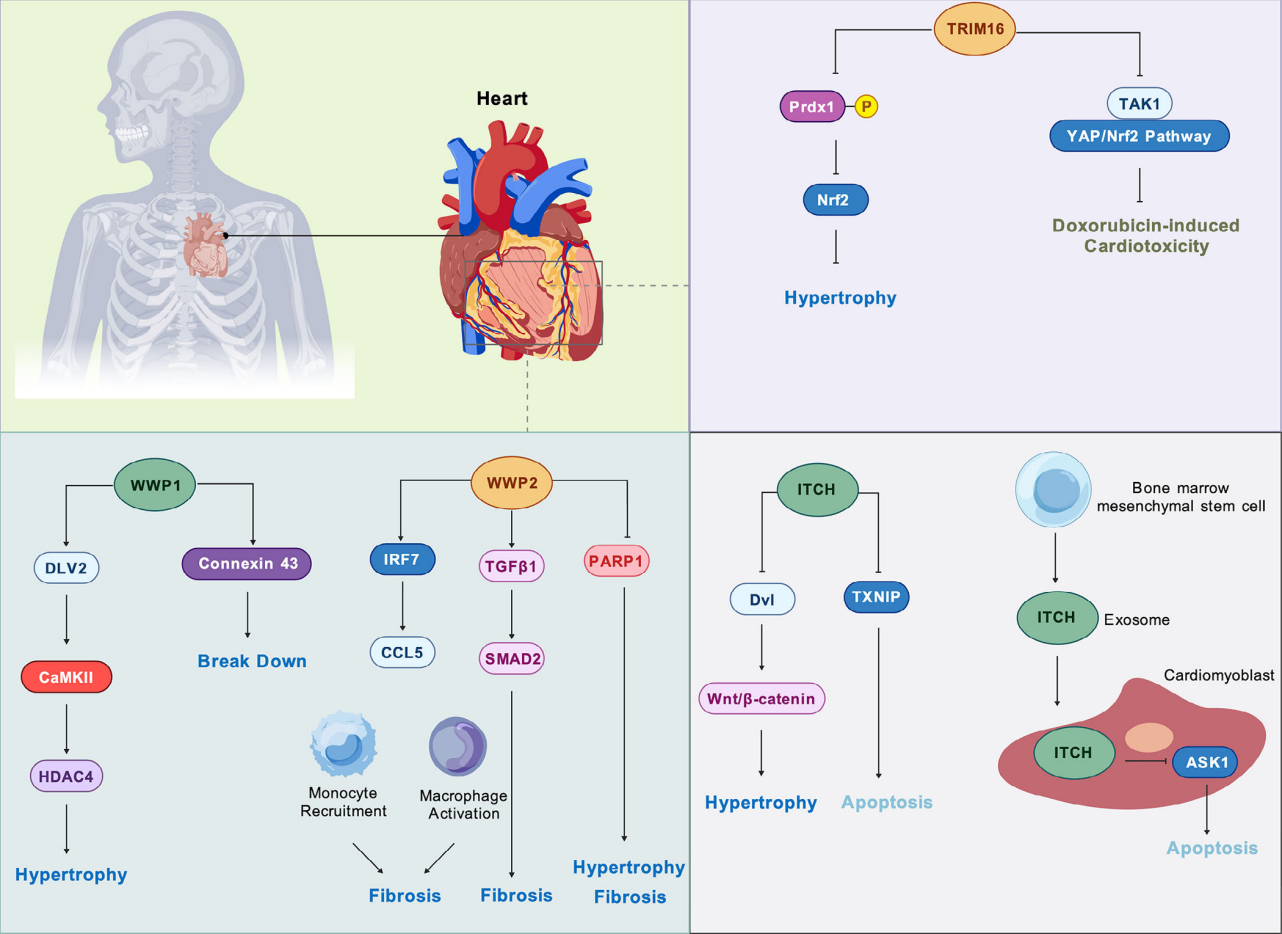
E3 ubiquitin ligases as key modulators of heart failure. (A) Dual roles of the WWP E3 ligase family WWP1 promotes hypertrophy via the DVL2–CaMKII–HDAC4 axis and degrades Connexin 43. WWP2 drives fibrosis by activating the IRF7–CCL5 inflammatory axis and enhancing TGFβ1/SMAD2 signaling. Conversely, WWP2 can limit hypertrophy and fibrosis by mediating PARP1 degradation. (B) Cardioprotective functions of TRIM16. TRIM16 suppresses hypertrophy by activating the Prdx1–Nrf2 antioxidant pathway. It also mitigates doxorubicin‐induced cardiotoxicity by modulating TAK1 and YAP/Nrf2 signaling. (C) Protective roles of ITCH. Intracellularly, ITCH prevents hypertrophy by degrading Dvl to inhibit Wnt/β‐catenin signaling and suppresses apoptosis by targeting TXNIP for degradation. In a paracrine manner, BMSC‐derived exosomes deliver ITCH to cardiomyoblasts, where it degrades the proapoptotic kinase ASK1 to attenuate apoptosis.

Heart failure (HF) and its common precursors, pathological cardiac hypertrophy and myocardial fibrosis, represent a significant global cardiovascular burden [231, 232]. These conditions often precede the development of clinically manifest heart failure, contributing significantly to morbidity and mortality worldwide [233]. E3 ligases are recognized as critical regulators of myocardial remodeling, oxidative stress, rhythm stability, and immune‐inflammatory responses associated with heart failure (Figure 4) [234, 235]. Specifically, E3 ligases have been shown to influence the synthesis and degradation of key proteins involved in these processes, thereby modulating cardiac function and structure [229, 236].

The WWP family belongs to C2–WW–HECT type E3 ligases and is divided into WWP1 and WWP2 [237, 238]. WWP1 is significantly upregulated in both the aortic constriction model and the hearts of patients with cardiac hypertrophy. Its activation enhances the DVL2/CaMKII/histone deacetylase (HDAC)4 signaling pathway through K27‐linked ubiquitin chain modification, thereby promoting left ventricular hypertrophy [239]. Additionally, WWP1 mediates the degradation of the cardiac gap junction protein connexin 43, which can lead to severe arrhythmias [240]. Genetic knockout of WWP1 significantly alleviated myocardial hypertrophy and dysfunction, suggesting its central regulatory role in pathological myocardial remodeling [241]. Inhibiting WWP1 may offer a new strategy for intervening in heart failure and arrhythmic disorders.

WWP2 is another member of the WWP family, and its architecture consists of a C2 domain at the N‐terminus, four WW repeats centrally, and a catalytic HECT domain at the C‐terminus [242]. In bone marrow‐derived cells, WWP2 activates the transcription factor IRF7 via monoubiquitination, thereby inducing CCL5 expression [243]. This signaling cascade facilitates the recruitment of Ly6c^high^ monocytes and the activation of macrophages, ultimately aggravating myocardial fibrosis. Moreover, in a hypertension‐induced nonischemic cardiomyopathy model, WWP2 deletion markedly reduced both fibrotic remodeling and cardiac dysfunction [243]. The E3 ubiquitin ligase WWP2 drives a conserved profibrotic gene network through its N‐terminal isoform by mediating TGFβ1/SMAD2 signaling [244]. In sepsis‐induced cardiac injury and heart failure, the E3 ubiquitin ligase WWP2 protects against cardiac remodeling by targeting poly (ADP‐ribose) polymerase‐1 (PARP1) for ubiquitination and proteasomal degradation. This action suppresses the excessive PARP1 activity and PARylation that drive myocardial hypertrophy and fibrosis, revealing a novel therapeutic axis for heart failure [245]. These findings emphasize the pivotal role of WWP2 is involved modulating the inflammation–fibrosis axis, suggesting that targeting WWP2 could possess potential antifibrotic properties.

E3 ubiquitin ligase TRIM16 is a critical regulator in cardiovascular pathologies, including myocardial hypertrophy, myocardial ischemia, and reperfusion injury, and exerts either significant cardiovascular protection or pathological change promotion functions [246, 247]. TRIM16 has recently been identified as a novel suppressor of pathological cardiac hypertrophy, acting through interaction with Prdx1 to inhibit its phosphorylation and thereby activate the Nrf2 pathway, ultimately attenuating cardiomyocyte enlargement and adverse remodeling [247]. The TRIM16–Prdx1 axis thus emerges as a potential therapeutic target for hypertrophy‐associated heart failure [247]. Moreover, TRIM16 can also alleviate doxorubicin‐induced cardiotoxicity by regulating the TAK1 and YAP/Nrf2 signaling pathways, exhibiting anti‐inflammatory and antioxidative stress effects [248]. In a chronic kidney disease‐related heart injury model, TRIM16 mediates the ubiquitination and degradation of RIP2, inhibits p38 phosphorylation, and alleviates myocardial hypertrophy and diastolic dysfunction [249].

ITCH, a HECT domain E3 ubiquitin ligase, has been shown to exert cardioprotective effects under various cardiac stress conditions [250]. In hypertrophy, ITCH mediates the ubiquitin–proteasomal degradation of Disheveled (Dvl) proteins, which consequently suppresses the prohypertrophic Wnt/β‐catenin signaling cascade [251]. Additionally, the E3 ubiquitin ligase ITCH is cardioprotective against ROS‐induced damage by targeting the thioredoxin inhibitor TXNIP for ubiquitin–proteasomal degradation [252]. This action enhances the cell's antioxidant capacity, thereby reducing cardiomyocyte apoptosis and preserving cardiac function following insults like doxorubicin administration or myocardial infarction (MI) [252]. Exosomes derived from bone marrow mesenchymal stem cells (BMSCs) protect against myocardial injury by delivering the E3 ubiquitin ligase ITCH into cardiomyoblasts [253]. In recipient cardiomyoblasts, the delivered ITCH attenuates apoptosis by mediating the ubiquitin–proteasomal degradation of its substrate, the proapoptotic kinase ASK1 [253]. Collectively, these findings highlight the dual role of ITCH in both mitigating cardiac hypertrophy and protecting cardiomyocytes, suggesting that its activation may provide a basis for developing combination therapeutic strategies for a range of cardiomyopathies.

### 4.2 Myocardial Infarction

MI and subsequent ischemia/reperfusion (I/R) injury are major contributors to the development of heart failure [254, 255, 256]. Parkin, a RBR type E3 ligase, primarily functions in marking damaged mitochondria for selective autophagy [257]. Parkin has been shown to protect against I/R injury by directly ubiquitinating cyclophilin‐D, a mechanism that inhibits mitochondrial permeability transition pore‐dependent necrosis [258]. Building on Parkin's protective role, direct pharmacological activation with the small molecule PR‐364 has been demonstrated to enhance mitophagy and mitochondrial metabolism, thereby preserving cardiac function and reducing mortality in a post‐MI setting [259]. Furthermore, natural compounds like berberine have been found to confer cardioprotection against pressure‐overload‐induced heart failure by upregulating the PINK1/Parkin signaling axis to restore suppressed mitophagy [260].

FBXL8, a key F‐box protein in the SCF E3 ligase complex, regulates fibrosis after MI [261, 262]. FBXL8 recognizes and facilitates the ubiquitination and degradation of the profibrotic transcription factor Snail1, inhibiting its induction of myofibroblast differentiation, reducing collagen deposition and cardiac remodeling [261]. Furthermore, FBXL8‐mediated downregulation of Snail1 also suppresses the RhoA signaling pathway, enhancing its antifibrotic effects [261]. FBXL8 knockout resulted in Snail1 accumulation, which significantly exacerbated myocardial fibrosis following infarction. These results suggest that the FBXL8‐Snail1 axis emerges as a critical regulator of myocardial repair and fibrosis progression [261].

The E3 ubiquitin ligase TRIM16 has been identified as a novel protective factor against myocardial I/R injury [246]. Mechanistically, TRIM16 promotes the K48‐linked polyubiquitination and subsequent degradation of NLRP3, thereby suppressing inflammasome activation, inflammation, and pyroptosis in cardiomyocytes [246]. This positions TRIM16 as a potential therapeutic target for treating MI [246]. Additionally, the E3 ligase MARCH2 has been identified as another crucial protective factor against myocardial I/R injury [263]. It suppresses cardiomyocyte pyroptosis by targeting the mitochondrial protein PGAM5 for K48‐linked ubiquitination and degradation, which disrupts the PGAM5–MAVS interaction and prevents activation of the NLRP3 inflammasome [263]. In a separate protective mechanism, the E3 ligase WSB1 was also shown to be upregulated and exert a cardioprotective effect during myocardial I/R injury [264]. It accomplishes this by targeting the signaling kinase GSK3β for ubiquitination and degradation, an action that removes a key inhibitor of the prosurvival β‐catenin pathway and restrains cardiomyocyte death [264]. In contrast, some E3 ligases can exacerbate cardiac injury. The expression of neuregulin receptor degradation protein‐1 (Nrdp1) was found to be markedly upregulated after I/R, where it increased infarct size, inflammation, and cardiomyocyte apoptosis [265]. This detrimental effect was attributed to Nrdp1‐mediated degradation of its substrate, the ErbB3 receptor, which led to the suppression of prosurvival AKT/ERK1/2 signaling and the activation of stress‐related pathways [265].

### 4.3 Atherosclerosis

Atherosclerosis (AS) is a cardiovascular disease driven by chronic inflammation of the arterial wall and lipid deposition [266]. Recent studies have revealed that various E3 ubiquitin ligases play critical regulatory roles in the initiation and progression of AS by modulating lipid metabolism, cell death, and vascular cell function.

RNF128, a RING‐type E3 ligase, is significantly upregulated in macrophages under hyperlipidemic conditions [267]. It mediates Lys63‐linked nondegradative ubiquitination of the macrophage scavenger receptor SR‐B1, preventing its lysosomal degradation and promoting its recycling to the cell membrane [267]. Consequently, RNF128 enhances the uptake of oxidized low‐density lipoprotein (ox‐LDL), contributing to cholesterol accumulation and foam cell formation, which ultimately exacerbates atherosclerotic lesions [267]. Macrophage‐specific knockout of RNF128 significantly slowed plaque formation, and human single‐cell RNA sequencing data revealed that high expression of RNF128 is associated with an increased accumulation of inflammatory macrophages in the arterial wall [267]. RNF128 represents a potential therapeutic target in AS, offering a mechanism to tune macrophage cholesterol uptake by modulating nondegradative ubiquitination.

ITCH, a HECT‐type E3 ligase involved in metabolic homeostasis and cell death, has emerged as a key mediator of ferroptosis in endothelial cells during AS [268, 269]. ox‐LDL stimulation induces ITCH to mediate the K48‐linked ubiquitination and subsequent proteasomal degradation of ferritin light chain (FTL) [269]. This degradation of FTL leads to intracellular free iron accumulation, thereby triggering ferroptosis and exacerbating endothelial cell injury [269]. Conversely, targeting ITCH through either genetic silencing or small‐molecule inhibition successfully mitigated ferroptosis, improved endothelial function, and ultimately alleviated atherosclerotic plaque development in *Apoe^−^/^−^* mice [269]. This finding emphasizes the central role of ITCH in regulating endothelial iron homeostasis and arterial barrier function, suggesting it as a novel intervention target for iron death‐related vascular injury.

TRAF6, a RING‐type E3 ligase involved in mediating TLR and NF‐κB signaling, has gained increasing attention for its role in vascular smooth muscle cells (VSMCs) [270, 271]. TRAF6 contributes to the instability of atherosclerotic plaques by targeting VSMCs [272]. Evidence indicates that TRAF6 mediates the ubiquitination and subsequent degradation of the key VSMC protein SM22α [272]. This loss of SM22α activates G6PD and disrupts the glutathione reductase system, which impairs the cell's antioxidant capacity and ultimately promotes apoptosis [272]. The resulting depletion of VSMCs weakens the plaque's fibrous cap, a critical factor that increases the risk of rupture and acute cardiovascular events [272, 273]. Therefore, TRAF6 acts as a crucial regulatory hub in advanced AS, not only by driving inflammation but also by directly compromising the structural integrity of the blood vessel.

### 4.4 Arrhythmias

Arrhythmias are rhythm disturbances caused by abnormal myocardial electrical activity, closely related to dysregulation of ion channel expression, organelle homeostasis, and stress signaling [274, 275]. Numerous studies have shown that specific E3 ubiquitin ligases play critical roles in maintaining cardiac rhythm by regulating ion channels or mitochondrial homeostasis through ubiquitination.

NEDD4L is an E3 ubiquitin ligase belonging to the HECT domain NEDD4 subfamily, it is widely expressed during development and has been shown to play an important role in the pathogenesis of various cardiovascular diseases by regulating multiple pathways [276, 277, 278]. In cardiomyocytes, the HECT‐type E3 ligase NEDD4L is a critical regulator of the cardiac voltage‐gated sodium channel, Nav1.5 [279]. The enzymatic activity of NEDD4L is itself controlled by autoregulatory intramolecular interactions involving its C2 domain and first WW‐linker [279]. Mechanistic studies have revealed that NEDD4L specifically ubiquitinates the cytoplasmic linker between the first and second transmembrane domains (DI‐DII) of Nav1.5, targeting it for degradation [280]. This degradative pathway is antagonized by the ubiquitin‐like protein FAT10, which stabilizes Nav1.5 by competitively binding to the channel [280]. Indeed, conditional deletion of FAT10 in cardiac myocytes reduces membrane Nav1.5 expression and peak Na⁺ currents, leading to prolonged ECG intervals and a higher susceptibility to ventricular arrhythmia after myocardial ischemia [280]. This cardioprotective effect occurs because FAT10 binds to the C‐terminus of Nav1.5, thereby sterically hindering the interaction with NEDD4L (NEDD4‐2) and preventing the channel's degradation [280]. These studies suggest that NEDD4L plays a “double‐edged sword” role in maintaining sodium channel homeostasis and electrophysiological stability, with its activity needing to be carefully regulated to prevent excessive or insufficient Nav1.5 expression.

The E3 ubiquitin ligase WWP2 and the protein phosphatase 1 regulatory subunit PPP1R3A exhibit a dynamic and inverse regulatory relationship during the progression of arrhythmia‐induced cardiac damage [281]. In a murine model of arrhythmia, PPP1R3A is significantly overexpressed during the initial stages, a period when WWP2 is only marginally upregulated. As the disease advances to a severe state, this pattern inverts and PPP1R3A expression is downregulated and becomes more restricted to the nucleus, while WWP2 becomes highly overexpressed throughout the cardiac tissue [281]. Further evidence supporting a counter‐regulatory mechanism comes from experiments where silencing PPP1R3A in the early phase of arrhythmia led to a marked increase in WWP2 expression [281]. These findings suggest that PPP1R3A may exert a protective effect in the initial stages of cardiac injury, possibly by restraining the activity of WWP2, which appears to be a key contributor to pathological tissue remodeling as the condition worsens [243, 281].

### 4.5 Cardiomyopathy

Cardiomyopathy encompasses a group of diseases characterized by structural and functional abnormalities of the myocardium, with common types including metabolic cardiomyopathy, geriatric cardiomyopathy, and fibrosis‐driven nonischemic cardiomyopathy [282, 283]. Recent studies have demonstrated that several E3 ubiquitin ligases play crucial roles in the pathogenesis and progression of cardiomyopathy by regulating mitochondrial homeostasis, inflammatory pathways, and myocardial remodeling processes.

Parkin, a classical E3 ligase that regulates mitophagy, plays a critical role in maintaining mitochondrial quality control [284]. In the context of obesity‐induced cardiomyopathy, a high‐fat diet impairs Parkin‐mediated mitophagy by downregulating its expression and reducing its recruitment to damaged mitochondria [285]. This pathological defect can be reversed by enhancing cardiac fatty acid oxidation, which restores Parkin localization and maintains mitochondrial quality control [285]. Similarly, the PINK1‐Parkin pathway is suppressed in diabetic cardiomyopathy, a defect that can be therapeutically targeted by agents such as canagliflozin, which activates Parkin‐dependent mitophagy to alleviate cardiac dysfunction [286]. Further evidence reveals that restoring Parkin function is critical for suppressing ferroptosis in the diabetic cardiac microvasculature. The drug nicorandil, for example, activates a mitochondria‐localized AMPK–Parkin–ACSL4 signaling pathway to promote mitophagy, which in turn inhibits the proferroptotic enzyme ACSL4 [287]. Beyond its canonical role in protective mitophagy, Parkin can also be recruited to pathological pathways. For instance, the obesity‐induced protein STX17, located at the mitochondria‐associated ER membranes, promotes the Parkin‐dependent degradation of the mitochondrial calcium uniporter inhibitor MCUb, thereby contributing to mitochondrial calcium overload and cardiac dysfunction [288].

MG53, also known as tripartite motif‐containing protein 72 (TRIM72), is a member of the TRIM family of E3 ubiquitin ligases and plays a crucial role as a membrane repair protein in myocytes [289, 290]. Functionally, MG53 supports the survival and repair of cardiomyocytes and fibroblasts, with numerous studies linking it to ischemic, septic, and hypertrophic forms of cardiomyopathy [291, 292]. In sepsis‐induced cardiomyopathy, MG53 enhances myocardial performance and attenuates structural injury, partly through exosome‐mediated circRNA regulation, whereas in hypertrophic cardiomyopathy, MG53 stabilizes potassium channel function to counteract arrhythmogenic remodeling [293, 294, 295]. Conversely, chronic elevation of MG53 has been associated with insulin resistance and disordered lipid metabolism, thereby contributing to diabetic cardiomyopathy [291, 296, 297]. To address this problem, novel approaches such as MG53 mutants and antibody‐based interventions have been proposed to dissociate its detrimental metabolic effects from its cardioprotective functions [297, 298]. Collectively, current evidence highlights MG53 as a context‐dependent regulator in cardiomyopathies and a promising target for therapeutic translation.

The E3 ligase WWP2 has been identified as a key regulator of cardiac fibrosis and dysfunction in cardiomyopathy [244]. It promotes fibrosis through a dual mechanism: by directly activating a profibrotic “ECM gene network” in myofibroblasts and by regulating a distinct population of profibrogenic Ly6C‐high monocytes via the IRF7–CCL5 axis in nonischemic cardiomyopathy [243]. Given its central role, targeting the WWP2 pathway is emerging as a promising therapeutic strategy. For instance, the bile acid ursodeoxycholic acid (UDCA) has demonstrated significant antifibrotic effects in cellular models and living human myocardial slices [299]. Crucially, in cardiac fibroblasts isolated from patients with dilated cardiomyopathy, UDCA treatment was found to successfully reverse the activity of the profibrotic TGFβ/WWP2 gene network. These findings position agonists that inhibit the WWP2 signaling axis, such as UDCA, as excellent candidates for treating cardiac fibrosis in various forms of cardiomyopathy [299].

## 5 The Role of E3 Ligases in Autoimmune and Inflammatory Diseases

Autoimmune and inflammatory diseases represent a broad spectrum of debilitating conditions characterized by the common pathological feature of a dysregulated and overactive immune response [300]. In autoimmunity, the immune system breaches self‐tolerance, mounting a targeted assault against the body's own tissues, as seen in diseases like rheumatoid arthritis (RA), lupus, and multiple sclerosis (MS) [301]. More broadly, chronic inflammatory disorders are driven by the inappropriate or excessive activation of inflammatory pathways, leading to persistent tissue damage [302].

The UPS is central to the regulation of these critical pathways, with E3 ubiquitin ligases serving as the master architects of the cellular immune response [303]. By attaching ubiquitin chains to specific substrate proteins, these enzymes dictate their fate, targeting them for degradation, altering their subcellular localization, or modulating their enzymatic activity [57]. These E3 ligases provide a crucial layer of regulation, establishing activation thresholds for immune signaling and ensuring the timely termination of inflammatory responses to prevent excessive tissue damage [52, 304]. Consequently, the dysregulation of specific E3 ligases, through genetic mutation or altered expression, can disrupt this delicate balance, leading to the sustained pathway activation that drives the pathogenesis of both autoimmune and inflammatory diseases.

### 5.1 Systemic Lupus Erythematosus

Systemic lupus erythematosus (SLE) is a chronic autoimmune disease characterized by immune system dysfunction, where the body's adaptive immune system mistakenly attacks its own tissues, resulting in inflammation and damage to various organs, such as the skin, kidneys, joints, and heart [305, 306, 307]. SLE is associated with the production of autoantibodies, particularly against nuclear components like double‐stranded DNA, which leads to the deposition of immune complexes and the activation of inflammatory pathways [308]. The pathogenesis of SLE is closely linked to a breakdown in immune tolerance, excessive activation of IFN‐I signaling, and immune complex accumulation, all of which play central roles in disease progression [309, 310]. These immune disturbances are often exacerbated by both genetic and environmental factors [311]. E3 ubiquitin ligases are crucial regulators of immune signaling pathways involved in the onset and progression of SLE. They control critical immune responses, including T cell activation, inflammatory cytokine production, and the clearance of damaged or apoptotic cells, all of which are dysregulated in SLE.

**FIGURE 5:**
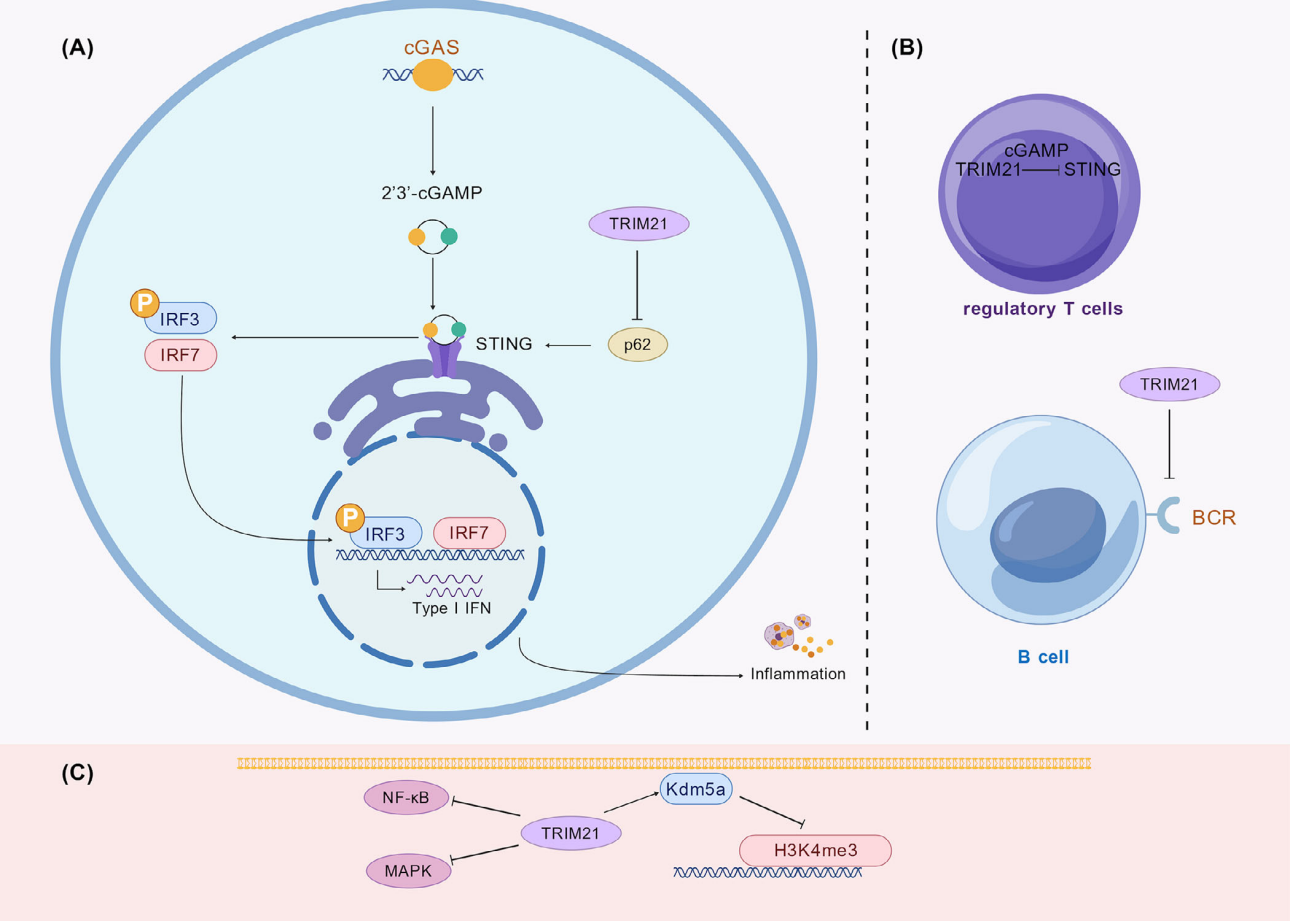
Mechanisms of immune dysregulation by E3 ligases in systemic lupus erythematosus (SLE). (A) TRIM21 potentiates the cGAS–STING pathway. Upon activation by cytosolic DNA, STING induces type I IFN production via IRF3/7 phosphorylation. TRIM21 sustains this signaling by preventing the p62‐mediated autophagic clearance of STING, exacerbating the inflammatory response. (B) TRIM21 function in lymphocytes. Dysfunction of TRIM21 impairs the negative regulation of the B‐cell receptor (BCR), promoting autoantibody production in B cells and contributing to the breakdown of immune tolerance. (C) Epigenetic suppression of A20 (TNFAIP3). In SLE, A20 is downregulated by the histone demethylase Kdm5a, which removes the activating H3K4me3 mark from the A20 promoter. The loss of this key negative regulator leads to hyperactivation of proinflammatory NF‐κB and MAPK signaling.

TRIM21 (Ro52), a tripartite motif‐containing E3 ubiquitin ligase, has emerged as a pivotal regulator at the intersection of autoimmunity, innate immune signaling, and malignancy [47]. Originally recognized as an autoantigen in SLE and primary Sjögren's syndrome, TRIM21 dysfunction has been linked to aberrant B‐cell differentiation, excessive autoantibody production, and IFN overactivation, thereby amplifying autoimmune pathology [312]. Mechanistic insights reveal that TRIM21 enhances cGAS‐STING pathway signaling by preventing autophagic clearance of STING through K63‐linked ubiquitylation of p62/SQSTM1, a process that stabilizes STING and sustains IFN production (Figure 5A) [313]. Clinically, the presence of anti‐TRIM21 autoantibodies correlates with reduced TRIM21 protein expression, elevated IFN‐β levels, and increased immunoglobulin subclasses, highlighting their role as potential biomarkers for immune dysregulation in SLE (Figure 5B) [312]. Beyond its immunoregulatory functions, TRIM21 exhibits dual roles in cancer, acting either as a tumor suppressor or promoter depending on the malignancy, and its altered expression in autoimmune populations has been implicated in lymphoma and breast cancer susceptibility [314]. Collectively, these findings indicate that TRIM21 serves as a central hub coordinating ubiquitin signaling, autophagy, and immune homeostasis, holding significant implications for the pathogenesis of autoimmune diseases.

Cbl‐b is a key E3 ligase in the regulation of adaptive immunity, primarily negatively regulating TCR signaling [315]. Recent findings have elucidated a key mechanism for Treg dysfunction in SLE, demonstrating that deficient expression of the E3 ubiquitin ligase Cbl‐b impairs the K63‐linked polyubiquitination of STAT‐3, leading to its hyper‐phosphorylation and the characteristic “resistance to suppression” phenotype [316]. Moreover, T cells from patients with SLE have been found to have reduced Cbl‐b expression, which corresponds with features of T‐cell hyper‐responsiveness. This functional defect is further underscored by a genetic link, as a specific Cbl‐b gene polymorphism has also been significantly associated with the disease [317]. The function of Cbl‐b proteins is also essential for B cell‐intrinsic checkpoints, as their dual deletion specifically in B lymphocytes leads to the development of a spontaneous SLE‐like disease [318]. This autoimmune phenotype was driven by defective B cell anergy, which resulted from a dysregulated B‐cell receptor (BCR) signalosome that failed to properly coordinate proximal activation signals with downstream adaptor protein function [318]. The T‐cell dysregulation in SLE has been linked to a broad deficit in anergy‐inducing factors, including the concurrent downregulation of Cbl‐b, GRAIL, and the transcription factor Egr2. Importantly, subsequent ex vivo studies demonstrated that rapamycin, in contrast to vitamin D, could successfully restore a stable and functionally suppressive Treg population from these patients over the long term.

A20, also known as TNFAIP3, is an immune negative regulator with dual functions as both a deubiquitinase (DUB) and an E3 ligase that acts as a critical negative regulator of inflammation and immune activation by inhibiting key signaling pathways such as NF‐κB and controlling cell death processes [319, 320]. A consistent finding across studies is that A20 expression is significantly downregulated in the immune cells of SLE patients, an insufficiency that correlates inversely with disease activity indices and the development of lupus nephritis [321]. This reduced expression is underpinned by complex genetic and epigenetic mechanisms. Fine‐mapping of the risk locus has identified functional variants, such as a TT>A (deletion T followed by a T to A transversion) polymorphic dinucleotide, located within distal enhancer elements [322, 323]. These risk alleles impair the binding of key transcription factors like NF‐κB, which disrupts the long‐range chromatin looping required for efficient interaction between the enhancer and the *TNFAIP3* promoter, ultimately attenuating A20 transcription [323]. This intricate regulatory network also involves other risk single nucleotide polymorphisms (SNPs) that can coordinately modulate the expression of *TNFAIP3* and adjacent inflammatory genes [324]. Complementing these genetic factors, A20 is also subject to epigenetic silencing in B cells via the histone demethylase Kdm5a, which diminishes the activating H3K4me3 mark at the A20 promoter (Figure 5C) [325].

The pathological consequences of A20 deficiency are diverse and extend beyond its canonical role as a negative regulator of NF‐κB [326]. Hyperactivation of NF‐κB and MAPK signaling, which drives B cell hyper‐proliferation and proinflammatory cytokine production, is a central outcome of A20 dysfunction, but other critical pathways are also involved [325]. Notably, genetic alterations specifically within A20's DUB domain promote SLE susceptibility through an NF‐κB‐independent mechanism involving the upregulation of PADI4, which enhances protein citrullination and the formation of neutrophil extracellular traps [327]. In organ‐specific manifestations, A20 deficiency in podocytes exacerbates lupus nephritis by dysregulating the NF‐κB/UCH‐L1 axis, while rare de novo mutations have been shown to impair A20's ubiquitin‐editing of TRAF6, potentially compromising the blood‐brain barrier in neuropsychiatric SLE [328]. The central role of this pathway is highlighted by preclinical evidence showing that therapeutic agents like daphnetin can ameliorate inflammation by upregulating A20, reinforcing its position as a promising therapeutic target for SLE [329].

### 5.2 Rheumatoid Arthritis

RA is a chronic autoimmune disease causing progressive, symmetric joint inflammation that leads to joint damage and disability. The disease typically advances from affecting a few joints to many, with systemic symptoms becoming common over time [330, 331, 332]. The pathogenesis of RA is complex, involving the activation of various immune pathways, including T cell differentiation, cytokine release, and chronic inflammation of the synovium [333, 334]. These processes are often accompanied by local immune imbalances and chronic inflammatory responses, which ultimately result in joint damage [333]. E3 ubiquitin ligases play a crucial role in regulating these immune and inflammatory responses [335]. Several E3 ubiquitin ligases participate in the immune dysregulation and synovial inflammation of RA, playing a central role in the disease.

The E3 ubiquitin ligase STIP1–homologous U‐Box containing protein 1 (STUB1) has recently emerged as a pivotal regulator of T cell‐mediated pathology in RA [336, 337, 338]. Overexpression of STUB1 is observed in RA patients, where it critically promotes the imbalance of proinflammatory Th17 cells over immunosuppressive regulatory T (Treg) cells [336]. Mechanistically, STUB1 achieves this by mediating the nondegradative, K63‐linked ubiquitination of the aryl hydrocarbon receptor, thereby skewing T cell fate [336]. Beyond the Th17/Treg axis, STUB1 also drives the expansion of pathogenic follicular helper T (Tfh) cells, another key cellular driver in RA [337]. In this context, STUB1 employs a distinct, degradative mechanism, targeting the scaffold protein p62 for K48‐linked ubiquitination, which in turn unleashes mTORC1 signaling to promote Tfh differentiation [337]. STUB1 disrupts multiple pathogenic T cell lineages through distinct ubiquitination pathways and modulates the immune microenvironment in RA, thereby emerging as a highly attractive therapeutic target for RA [337].

Ubiquitin‐like containing PHD and RING finger domains 1 (UHRF1), an E3 ligase with epigenetic regulatory functions, acts as a paradoxical negative regulator of synovial inflammation in RA [339]. Despite being upregulated in the synovial fibroblasts of arthritic joints, UHRF1 functions as a crucial suppressive factor, maintaining DNA methylation homeostasis through the recruitment of DNMT1 [339, 340, 341]. Translating these findings to human disease, UHRF1 expression levels in patient synovium are negatively correlated with clinical disease activity (DAS28), Th17 cell accumulation, and resistance to DMARD treatment, suggesting that insufficient UHRF1 function contributes to more severe and refractory disease [339]. Consequently, UHRF1 has emerged as a novel therapeutic target, with proof‐of‐concept studies demonstrating that its pharmacological stabilization by compounds like Ryuvidine can ameliorate arthritis in preclinical models, validating a new strategy aimed at reinforcing endogenous suppressive epigenetic mechanisms [339, 342].

The RING‐type E3 ligase Midline‐1 (Mid1) promotes pathological synovial cell proliferation and migration, a key feature of synovial activation in RA [343, 344]. Mechanistically, Mid1 exerts its proarthritic effects by targeting the enzyme dipeptidyl peptidase‐4 for ubiquitin‐mediated proteasomal degradation [343]. Beyond its impact on synovial fibroblasts, Mid1 has also been implicated in the inflammatory polarization of myeloid cells, where its expression appears to be regulated downstream of the Fas signaling pathway and contributes to joint degradation [344].

### 5.3 Inflammatory Bowel Disease

Inflammatory bowel disease (IBD), which includes Crohn's disease (CD) and ulcerative colitis (UC), is a group of diseases characterized by chronic inflammation of the gastrointestinal tract [345, 346]. The pathogenesis of IBD involves a combination of innate immune dysregulation, disruption of the epithelial barrier, and imbalance of the gut microbiota [347]. Recent studies have identified several E3 ubiquitin ligases as key regulators in the development of IBD, where they influence immune receptor signaling, epithelial homeostasis, and the microbiota–metabolism axis.

The E3 ubiquitin ligase RING finger protein 186 (RNF186), identified within IBD susceptible loci, has emerged as a multifaceted regulator of intestinal homeostasis [348, 349, 350]. In the colonic epithelium, RNF186 maintains gut integrity by controlling protein homeostasis, mitigating endoplasmic reticulum (ER) stress, and promoting autophagy through the ubiquitination of the EPHB2 receptor [348, 350]. Within the myeloid compartment, RNF186 is essential for innate immune responses, as it ubiquitinates the ER stress sensor ATF6 to facilitate signaling downstream of pattern recognition receptors (PRRs), leading to effective bacterial clearance [351, 352]. Human genetic studies, however, present a more complex role for RNF186 in IBD. While several IBD‐associated risk variants have been characterized as loss‐of‐function alleles that impair PRR‐initiated antimicrobial pathways, a rare protein‐truncating variant (p.R179X) that also results in a loss of function has been robustly shown to confer strong protection against UC [349, 351, 352, 353].

The membrane‐associated E3 ubiquitin ligase MARCH2 has been identified as a critical homeostatic regulator in the context of IBD [354]. The E3 ubiquitin ligase MARCH2 demonstrates a protective function in experimental colitis, where its absence leads to hyper‐inflammation and impaired intestinal barrier function. This protective role is attributed to its function as a negative regulator of the NF‐κB pathway, which it achieves by targeting the essential adaptor protein NEMO for proteasomal degradation [354, 355, 356, 357]. The mechanism of MARCH2 activation during inflammation has recently been detailed [354]. In a resting state, MARCH2 is held inactive through a direct interaction with another family member, MARCH8 [354]. Upon inflammatory stimulation, such as by tumor necrosis factor (TNF), this inhibitory interaction is disrupted, allowing MARCH2 to dimerize and undergo K63‐linked autoubiquitination at key lysine residues (K127 and K238) [354]. This autoubiquitination is the critical final step that activates MARCH2, enabling it to recognize and degrade NEMO, thereby providing a negative feedback loop to suppress excessive inflammatory signaling [354].

The E3 ligase ankyrin repeat and SOCS box‐containing protein 3 (ASB3) has recently been identified as a proinflammatory regulator in IBD, linking innate immune signaling in intestinal epithelial cells to the gut microbiota [358, 359]. Its expression is significantly upregulated in the intestinal lesions of IBD patients, and its absence confers resistance to colitis, which is marked by reduced NF‐κB activation and proinflammatory cytokine production [359]. Mechanistically, ASB3 targets the signaling adaptor TRAF6 for K48‐linked ubiquitination and degradation, and promotes the inflammatory response in the gut epithelium in a microbiota‐dependent manner, indicating its pathogenic effects are intertwined with microbial dysbiosis [359].

### 5.4 Multiple Sclerosis

MS is an autoimmune disease characterized by central nervous system (CNS) demyelination and chronic neuroinflammation, most commonly affecting young adults [360]. Research indicates that E3 ubiquitin ligases play important roles in the pathogenesis and immune regulation of MS. The ER‐resident E3 ligase Hrd1 (SYVN1), for instance, is highly expressed in T cells from MS patients. Hrd1 mediates the ubiquitination and degradation of the cell cycle inhibitor p27^KIP1^, thereby promoting T cell proliferation and activation [361]. As a positive regulator of T cell autoimmunity, Hrd1 upregulation appears to drive MS progression by enhancing pathogenic Th1 and Th17 responses [361].

The HECT E3 ligase NEDD4 promotes Th17‐mediated neuroinflammation by directly targeting the master transcription factor RORγt for nondegradative, K27‐linked polyubiquitination [362]. This modification enhances RORγt's transcriptional activity to drive pathogenic Th17 cell differentiation, and accordingly, T cell‐specific NEDD4 deficiency ameliorates experimental autoimmune encephalomyelitis (EAE), identifying it as a key propathogenic factor in MS [362]. Conversely, the RING E3 ligase RNF157 acts as a negative regulator of neuroinflammation, whose expression is downregulated in T cells from MS patients and negatively correlates with levels of the master Th17 transcription factor, RORγt [363, 364]. Mechanistically, RNF157 mediates the K48‐linked polyubiquitination and degradation of HDAC1, a process that modulates the epigenetic landscape of Th17‐related genes to restrain Th17 polarization [363]. Therefore, the downregulation of RNF157 in MS may dismantle this regulatory brake, leading to an excessive Th17 response [363].

The E3 ubiquitin ligase RNF213 promotes the differentiation of protective Treg cells by mediating the K63‐linked ubiquitination and nuclear translocation of the transcription factor FOXO1 [365]. This immunomodulatory axis is highly relevant to MS, as RNF213 is induced by the therapeutic agent IFN‐β and is essential for its clinical efficacy, thus providing novel insight into the drug's mechanism of action [365, 366]. Moreover, the E3 ubiquitin ligase Mid1 regulates the migration of effector T cells to the CNS via the mTOR/microtubule pathway during neuroinflammation [367]. Consistent with this function, Mid1 is upregulated in the EAE model, where its T cell‐specific deletion impairs immune cell infiltration of the CNS and ameliorates disease, highlighting it as a novel therapeutic target for MS [367].

The E3 ubiquitin ligase Cbl‐b, a crucial negative regulator of T cell activation, is expressed at reduced levels in CD4⁺ T cells from MS patients during relapse [368, 369]. This deficiency is linked to the MS‐associated genetic risk variant rs12487066, which enhances binding of the repressive transcription factor C/EBPβ to the *CBLB* gene, thereby lowering Cbl‐b expression, impairing immune tolerance, and altering T cell responses to viral triggers [368]. Functionally, this loss of Cbl‐b unleashes the production of the pathogenic cytokine GM‐CSF from T cells [370]. The exacerbated EAE in Cbl‐b knockout mice is GM‐CSF‐dependent and is driven by increased binding of the NF‐κB p50 subunit to the GM‐CSF promoter [370]. This establishes a key pathway through which Cbl‐b deficiency contributes to autoimmunity by permitting T‐cell mediated pathology [370].

The integrity of the blood–spinal cord barrier (BSCB) is also governed by E3 ligase activity [371]. The chemokine CXCL13, found at elevated levels in MS, has been shown to disrupt the BSCB by inducing excessive endothelial cell autophagy [371]. This pathogenic process is driven by the CXCL13‐mediated upregulation of the E3 ligase RNF6, which then targets the autophagy receptor SQSTM1 for ubiquitination, offering a new therapeutic axis for protecting the CNS [371].

Beyond immune cells, E3 ligases also regulate the pathogenic activities of CNS‐resident cells [372, 373]. TRIM21 has emerged as a key regulator of astrocyte metabolism. In reactive astrocytes, upregulated TRIM21 promotes the nuclear translocation of the glycolytic enzyme PKM2 [373]. Within the nucleus, PKM2 functions as a transcriptional coactivator for proinflammatory pathways (e.g., STAT3 and NF‐κB), orchestrating a metabolic shift toward glycolysis and augmenting inflammatory cytokine production [373]. Crucially, suppression of the TRIM21–PKM2 axis via pharmacological or genetic means reduced disease severity in the EAE model, indicating that the TRIM21–PKM2 axis represents a promising therapeutic target [373]. Moreover, the monocarboxylate transporter MCT4, which promotes astrocyte reactivity via a nonmetabolic mechanism, is pathologically stabilized and upregulated in the EAE model due to the downregulation of its E3 ubiquitin ligase, TRIM7 [374, 375]. Significant improvement in EAE was achieved through astrocyte‐specific knockdown of MCT4, confirming its propathogenic role and thereby identifying the TRIM7–MCT4 axis as a novel therapeutic target for MS [375].

### 5.5 Autoimmune Thyroid Diseases

Autoimmune thyroid diseases (AITD), including Graves’ disease (GD) and Hashimoto's thyroiditis (HT), are organ‐specific autoimmune disorders that primarily affect women and are characterized by immune‐mediated damage to the thyroid gland [376, 377]. Recent studies have revealed that various E3 ubiquitin ligases play a critical role in AITD, primarily centered on how their dysfunction can lead to the breakdown of immune tolerance.

c‐Cbl, a classic RING‐type E3 ligase, is significantly downregulated in peripheral blood mononuclear cells from GD patients [378]. CBL mRNA expression is significantly reduced in GD patients, particularly in relapsing cases, and negatively correlates with clinical severity markers like thyroid enlargement and thyrotropin receptor antibody levels, marking it as a potential biomarker for disease recurrence [378].

Genetic variations affecting the anti‐inflammatory ubiquitin‐modifying enzyme A20 (encoded by *TNFAIP3*) have been identified as specific risk factors for GD [379]. A study in a Chinese Han population linked several *TNFAIP3* SNPs to GD risk, an association that was notably absent in HT, highlighting a distinct role for A20 in the pathogenesis of GD [379].

### 5.6 Psoriasis

Psoriasis is a chronic, immune‐mediated inflammatory skin disorder characterized by hyperkeratosis, T cell infiltration, and abnormal differentiation of epidermal cells [380, 381]. Recent research highlights the significant role of E3 ubiquitin ligases in the pathogenesis of psoriasis by regulating key inflammatory and proliferative pathways.

RNF114 (also known as ZNF313) is a RING‐type E3 ligase identified as a psoriasis susceptibility gene through multiple genomic studies [382]. A genome‐wide association scan for psoriasis identified a novel susceptibility locus on chromosome 20q13, pinpointing the E3 ubiquitin ligase RNF114 as the likely causal gene [383]. The disease‐associated SNP, rs495337, functions as a regulatory variant that increases RNF114 expression in skin and immune cells, suggesting that overexpression of this immune‐regulatory E3 ligase contributes to psoriasis pathogenesis [383]. Functionally, RNF114 has been shown to operate within a positive feedback loop that amplifies the innate antiviral RIG‐I/MDA5 signaling pathway [382]. By enhancing NF‐κB and IRF3 activity, RNF114 boosts the production of IFN‐I, suggesting that its genetic dysregulation in psoriasis contributes to pathogenesis by driving excessive inflammatory cytokine production in the skin [382].

A20 and its cofactor ABIN1 (TNIP1) form a widely recognized core of ubiquitin pathway regulation in psoriasis [384]. A20 robustly suppresses both IL‐17 and TNF‐α‐induced gene programs, whereas ABIN1 exerts a much more modest effect [384]. Crucially, the inflammatory genes targeted by A20 are aberrantly upregulated in the epidermis across a wide range of rashes, including psoriasis and atopic dermatitis, implicating the dysregulation of this keratinocyte‐intrinsic pathway as a common pathogenic feature in skin inflammation [384]. Moreover, studies show that a partial deficiency of ABIN‐1 leads to a hyper‐responsive state characterized by increased expression of viral sensors (e.g., RIG‐I, MDA5) through an NF‐κB‐dependent, RIPK1‐independent mechanism [385]. Furthermore, this sensitization involves a RIPK1 kinase‐dependent pathway for proinflammatory cytokine production, revealing a dual‐layered regulatory control by ABIN‐1 over innate immunity [385].

The HECT E3 ligase NEDD4L acts as a negative regulator of psoriasis by inhibiting the IL‐6/GP130 signaling pathway directly within keratinocytes [386]. NEDD4L mediates the K27‐linked ubiquitination and proteasomal degradation of the GP130 receptor, and its downregulation in psoriatic skin correlates with increased inflammatory signaling, highlighting a novel therapeutic axis [386].

The propathogenic factor fibroblast growth factor 12 (FGF12), which is highly expressed in psoriatic lesions, promotes keratinocyte hyperproliferation by suppressing the p53 signaling pathway [387]. Mechanistically, FGF12 binds to the E3 ligase MDM2 and prevents its β‐Trcp‐mediated ubiquitination and degradation; the resulting stabilization of MDM2 leads to p53 suppression, identifying FGF12 as a novel therapeutic target [387].

A psoriasis‐causing mutation, CARD14E138A, drives inflammation through two separate downstream pathways. It activates NF‐κB and MAP kinases in a manner dependent on the E3 ligases HOIP and TRAF6—a process negatively regulated by A20 and ABIN1; concurrently, it activates mTORC1 to drive metabolic changes in keratinocytes. Crucially, blocking the mTORC1 pathway with rapamycin is sufficient to reduce keratinocyte proliferation and skin pathology, identifying mTORC1 inhibition as a promising therapeutic approach for this form of psoriasis [388].

The TRIM family of E3 ubiquitin ligases plays a multifaceted role in the pathogenesis of psoriasis, with different members acting as either drivers or suppressors of inflammation and keratinocyte hyperproliferation. Several TRIM proteins function as propathogenic factors. TRIM21, which is upregulated in psoriatic skin, promotes inflammation through two distinct mechanisms [389]. First, it directly ubiquitinates the NF‐κB subunit p65 via a K63‐linkage, enhancing its phosphorylation and nuclear activation [389]. Second, it ubiquitinates and stabilizes keratin 17, which in turn promotes the activation of the proproliferative transcription factor STAT3 [390]. Similarly, TRIM33 is also upregulated and contributes to the disease through dual actions. It enhances inflammation by ubiquitinating Annexin A2, which promotes the nuclear retention and activity of NF‐κB [391]. Concurrently, it drives metabolic reprogramming and cell growth by ubiquitinating the enzyme PKM2, leading to its nuclear retention and subsequent activation of STAT3 [392]. Notably, both TRIM21 and TRIM33 converge on activating the key psoriatic transcription factors NF‐κB and STAT3, albeit through different substrates [389, 390, 391, 392].

In contrast, other TRIM members act as crucial brakes on inflammation within the psoriatic microenvironment [393]. TRIM15 functions as a negative feedback regulator induced by TNF‐α. It dampens the NF‐κB pathway by inhibiting the ubiquitination of the upstream kinase TAK1 and by antagonizing the proinflammatory function of TRIM8, with which it shows an inverse correlation in psoriatic conditions [393]. Collectively, these findings demonstrate that the balance between pro‐ and anti‐inflammatory TRIM E3 ligase activity is critical for skin homeostasis. Their dysregulation in psoriasis suggests this family of enzymes may offer novel therapeutic targets.

### 5.7 Gout

Gout is an acute, nonseptic arthritis induced by monosodium urate (MSU) crystals, with its core pathogenesis involving NLRP3 inflammasome activation and massive release of IL‐1β [394, 395, 396]. E3 ubiquitin ligases and related modifying enzymes are key regulators of the inflammatory cascade in gout, primarily by controlling the activation of the NLRP3 inflammasome and subsequent pyroptosis [397, 398, 399]. Several E3 ligases act as direct drivers of the disease, MDM2 is upregulated in gouty arthritis, where it mediates the ubiquitination and degradation of the protective nuclear receptor PPARγ, thereby unleashing NLRP3 activation [397]. Similarly, the mitochondrial E3 ligase MARCH5 is induced by MSU crystals and promotes inflammation by ubiquitinating SIRT3, leading to mitochondrial dysfunction [400]. The inflammatory signaling is further amplified by the activation of the TRAF6–TAK1 axis, a process sustained by MSU crystals’ ability to lock TAK1 in an active state, while the protective DUB A20 is simultaneously inhibited [401].

Conversely, the E3 ligase NEDD4, normally a protective factor that degrades the inflammatory sensor NOD1, becomes inhibited by S‐nitrosylation in gout. This modification disables NEDD4's E3 ligase activity, leading to an accumulation of NOD1 and exacerbated pyroptosis [398]. Other E3 ligases regulate the NLRP3 inflammasome through noncanonical, enzyme‐independent mechanisms. HECTD3 acts as an inhibitor by physically binding to NLRP3 and blocking its interaction with NEK7, a function independent of its E3 ligase activity [402]. Conversely, TRIM50 paradoxically promotes NLRP3 activation by binding to it and inducing its oligomerization, which prevents inhibitory ubiquitination [399].

## 6 Pathological Role of E3 Ligases in Metabolic Diseases

The increasing global prevalence of metabolic diseases, such as obesity, type 2 diabetes mellitus (T2DM), and nonalcoholic fatty liver disease (NAFLD), represents a formidable public health challenge [403]. Metabolic syndrome and its components are now highly prevalent, with global rates of central obesity, hypertension, and dyslipidemia each affecting over 40% of adults in some regions [404]. At the molecular level, the UPS serves as a critical regulator of cellular homeostasis. Within this system, E3 ubiquitin ligases function as pivotal enzymes that determine substrate specificity for protein ubiquitination and degradation, thereby modulating diverse cellular processes such as metabolic regulation, stress responses, and signal transduction (Table 1) [405, 406]. In recent years, the pathological role of E3 ligases in metabolic diseases has garnered significant attention. Multiple studies indicate that aberrant expression or functional dysregulation of E3 ligases is closely linked to various metabolic disorders, such as obesity, diabetes, and fatty liver disease [407, 408].

**TABLE 1:** The pathological roles of E3 ubiquitin ligases in metabolic diseases.

Category	E3 ligase	Role/function	Mechanism	Reference(s)
Glucose metabolism	CRL7	Negative regulator of insulin signaling	It targets insulin receptor substrate 1 (IRS1) for degradation, especially after mTORC1/S6K1 activation, thus attenuating insulin signaling	[409, 410]
	TRIM72 (MG53)	Multifaceted negative regulator of insulin action	Intracellularly, it targets the insulin receptor (IR) and IRS1 for degradation. Extracellularly, it is secreted as a myokine that blocks the IR, causing systemic insulin resistance.	[296, 297, 411, 412]
	TRIM32	Contributes to type 2 diabetes pathogenesis	In the liver, it drives insulin resistance by degrading the insulin receptor. In pancreatic β‐cells, it impairs insulin secretion and promotes autophagy.	[413, 414]
	Cbl family (c‐Cbl, Cbl‐b)	Dual role in insulin signaling	It acutely promotes GLUT4 translocation. Chronically acts as a negative regulator by targeting the insulin receptor for degradation, thereby increasing insulin sensitivity when absent.	[415, 416]
	FBXO28	Promotes pancreatic β‐cell survival	It protects pancreatic β‐cells from apoptosis, helping to preserve β‐cell mass. Its expression is reduced in diabetic conditions.	[417]
Dyslipidemia and homeostasis	MARCH6	Critical checkpoint in cholesterol homeostasis	It targets key cholesterol synthesis enzymes for degradation in a sterol‐dependent manner to regulate cholesterol production.	[418]
	Siah2	Essential regulator of adipogenesis	It orchestrates adipogenesis by degrading the antiadipogenic factor Zfp521 and creates a feedback loop by targeting PPARγ for degradation.	[419]
	RNF20	Coordinates adipose thermogenesis	It regulates thermogenesis tissue‐specifically: stabilizes GABPα in brown fat and promotes beiging in white fat by degrading NCoR1 (prolonged cold).	[420]
	TRIM56	Positive regulator of adipocyte browning	It promotes white adipose tissue browning by mediating the degradation of the corepressor TLE3, thereby activating the thermogenic program.	[421]
	RNF34	Negative regulator of thermogenesis	It negatively regulates thermogenesis by targeting the master coactivator PGC‐1α for degradation. Cold exposure suppresses RNF34 to stabilize PGC‐1α.	[422]
	FBXW7	Tissue‐specific controller of lipid metabolism	It suppresses thermogenesis in fat by degrading S6K1, protects the liver from steatosis, and suppresses adipocyte differentiation by degrading C/EBPα.	[423, 424]
	ITCH	Suppresses adipogenesis and maintains BCAA catabolism	It suppresses adipogenesis by ubiquitinating key lipogenic transcription factors; also maintains branched‐chain amino acid (BCAA) catabolism.	[425]
	FBXW2	Pathogenic/negative regulator of BCAA catabolism	It targets key BCAA catabolism enzymes for degradation, leading to BCAA accumulation, mTORC1 hyperactivation, and insulin resistance.	[426]
Mitochondrial function	Parkin	Central executor of mitochondrial quality control (mitophagy)	It orchestrates mitophagy by ubiquitinating outer membrane proteins on damaged mitochondria, which recruits autophagy adaptors for organelle clearance.	[427, 428]
	MUL1	Regulates mitochondrial dynamics and integrity	It regulates mitochondrial dynamics by degrading fusion protein Mfn2 (ubiquitin ligase) and stabilizing fission protein Drp1 (SUMO ligase).	[429, 430]
	MARCH5 (MITOL)	Governs mitochondrial quality control and dynamics	It regulates mitochondrial dynamics, mainly by degrading fission proteins (e.g., Drp1, Fis1); also modulates mitophagy and ER–mitochondria contacts.	[431, 432, 433]

### 6.1 E3 Ligases in Glucose Metabolism Disorders

Systemic glucose homeostasis is maintained through a complex interplay between insulin secretion from pancreatic β‐cells and insulin action in peripheral tissues, primarily skeletal muscle and adipose tissue, which are responsible for the majority of postprandial glucose disposal [434, 435, 436]. E3 ligases can attenuate insulin signaling by directly degrading key molecules like the insulin receptor (IR), or by indirectly regulating the pathway via proinflammatory mediators [437]. Furthermore, E3 ligases play a vital role in maintaining the health and mass of the insulin‐producing β‐cells themselves [438].

E3 ligases primarily induce insulin resistance by driving ubiquitin‐mediated degradation of key upstream components of the insulin signaling cascade, with the IR and IR substrate proteins serving as primary targets [409, 439]. Furthermore, E3 ligases indirectly regulate insulin signaling molecules by targeting proinflammatory mediators, which can impair the regulatory function of the insulin signaling pathway [437].

Cullin–RING E3 ubiquitin ligase 7 (CRL7) functions as a negative regulator of insulin signaling and glucose homeostasis [409, 440]. The absence of CRL7 enhances insulin sensitivity, cellular glucose uptake, and the activation of downstream PI3K/AKT signaling by preventing the degradation of IRS1 [409]. Mechanistically, CRL7 mediates the ubiquitination and degradation of IRS1 as part of a negative feedback loop triggered by hyperactivated mTORC1/S6K1 signaling [410]. This process requires S6K1‐mediated phosphorylation of multiple serine residues on the N‐terminus of IRS1, including Ser‐307/312 and Ser‐527, which creates a degradation signal recognized by CRL7 [410]. The N‐terminal region of IRS1 is also crucial for its interaction with CRL7 to ensure efficient ubiquitination [410].

Members of the TRIM family are also prominent regulators of insulin signaling. TRIM72(MG53) has emerged as a multifaceted negative regulator of insulin action through dual intracellular and extracellular mechanisms [289, 296, 297]. Intracellularly, MG53 functions as a muscle‐specific E3 ubiquitin ligase that targets both the IR and IRS1 for ubiquitin‐dependent degradation, thereby directly attenuating the signaling cascade within skeletal muscle [296]. This E3 ligase activity is dependent on the E2‐conjugating enzyme UBE2H [296, 439].

Beyond its intracellular role, MG53 also functions as a myokine secreted from muscle tissue in response to high glucose and insulin [297]. The secreted, circulating form of MG53 induces systemic insulin resistance by binding to the extracellular domain of the IR, where it acts as an allosteric blocker [297]. Clinically, elevated serum MG53 levels are associated with impaired glucose tolerance and β‐cell dysfunction in humans and serve as an independent predictor for the progression to type 2 diabetes [441]. Moreover, pharmacological disruption of the MG53–IRS1 interaction with small‐molecule inhibitors prevents IRS1 degradation, thereby enhancing insulin signaling in muscle cells and improving glucose homeostasis in animal models of insulin resistance [411, 412]. Considering the dual role of MG53 as a critical link between muscle metabolism and systemic glucose homeostasis, it holds significant potential for treating metabolic disorders [297, 411, 412].

TRIM32 has been identified as a critical E3 ubiquitin ligase contributing to type 2 diabetes pathogenesis through distinct, tissue‐specific mechanisms [413]. In the liver, high‐fat diet‐induced TRIM32 expression leads to proteasomal degradation of the IR, thereby driving hepatic insulin resistance [413]. Concurrently, in pancreatic β‐cells, high glucose elevates TRIM32 levels, leading to impaired insulin secretion and increased autophagy via inhibition of the Akt/mTOR pathway. This dual role is supported by clinical findings that circulating TRIM32 levels are significantly elevated in patients with T2DM [414].

The Casitas B‐lineage lymphoma (Cbl) family of proteins, including c‐Cbl and Cbl‐b, plays a complex dual role in insulin signaling [415]. They act as acute signal transducers in PI3K‐independent pathways. Upon insulin stimulation, they undergo tyrosine phosphorylation and translocate as a Cbl/CAP complex to the plasma membrane, a critical step for GLUT4 translocation [415]. Moreover, c‐Cbl functions as a chronic negative regulator and E3 ubiquitin ligase [415]. Mice lacking c‐Cbl show a profound increase in IR levels in skeletal muscle, leading to markedly improved whole‐body insulin sensitivity, increased energy expenditure, and a lean phenotype [416].

F‐box protein 28 (FBXO28), an SCF E3 ligase component, specifically promotes pancreatic β‐cell survival but does not regulate its function [417, 442]. FBXO28 expression is reduced in diabetic conditions, and its restoration protects β‐cells from death without impacting insulin content or secretion [417]. These findings suggest FBXO28 as a potential therapeutic target for preserving β‐cell mass in diabetes [417].

### 6.2 E3 Ligases in Dyslipidemia and Metabolic Homeostasis

MARCH6 is an ER‐resident E3 ligase that serves as a critical posttranslational checkpoint in cellular cholesterol homeostasis [418]. It directly targets the rate‐limiting enzymes of cholesterol biosynthesis, HMG‐CoA reductase (HMGCR) and squalene monooxygenase (SQLE), for ubiquitin‐mediated proteasomal degradation [418, 443]. This activity is exquisitely sensitive to cellular sterol levels, when cholesterol is abundant, the ER sensor protein Insig recruits MARCH6 to HMGCR and SQLE to initiate their degradation. Conversely, low cholesterol levels inhibit this interaction, stabilizing the enzymes to promote synthesis [418]. The MARCH6–Insig axis provides a rapid mechanism for regulating cholesterol production that complements the slower transcriptional control mediated by the SREBP pathway [443]. Moreover, MARCH6 also functions as an endogenous inhibitor of SREBP activity, as its deletion enhances the expression of SREBP target genes [443]. Loss of MARCH6 leads to cholesterol accumulation and has been implicated in the pathophysiology of NAFLD and AS [443].

The E3 ligase Siah2 regulates multiple stages of adipogenesis, including the initial commitment of progenitor cells and the activity of mature adipocytes. In the early stages of adipogenesis, Siah2 acts upstream of BMP‐4 to promote the degradation of the antiadipogenic transcription factor Zfp521 [419, 444]. This degradation is a critical step that allows for the expression of the proadipogenic factor Zfp423, which in turn drives the expression of PPARγ, the master regulator of adipocyte differentiation [409]. In addition to promoting its expression, Siah2 also directly targets the PPARγ protein itself for ligand‐dependent ubiquitination and degradation, creating a crucial feedback mechanism to modulate its activity in differentiated adipocytes [445]. Consequently, the loss of Siah2 impairs adipogenesis and leads to unhealthy adipocyte hypertrophy, underscoring its vital role in maintaining functional adipose tissue [409].

Beyond storing energy, adipose tissue can also dissipate it as heat through a process called nonshivering thermogenesis, primarily carried out by brown adipose tissue (BAT) and inducible “beige” or “brite” adipocytes within white adipose tissue (WAT) [446, 447]. The E3 ubiquitin ligase RNF20 orchestrates a sequential thermogenic response by acting distinctly in BAT and inguinal WAT (iWAT) [420]. Upon acute cold exposure, RNF20 is rapidly downregulated in BAT, which stabilizes its substrate GABPα to quickly boost thermogenic activity [420]. Conversely, during prolonged cold, RNF20 is gradually upregulated in iWAT, where it targets a different substrate, the corepressor NCoR1, for degradation [420]. This activates PPARγ and drives the formation of new beige adipocytes [420].

The E3 ubiquitin ligase TRIM56 is a key positive regulator of adipocyte browning and thermogenesis [421]. Its expression is upregulated by cold exposure, and its overexpression protects mice from diet‐induced obesity and helps maintain core body temperature [421]. Mechanistically, TRIM56 promotes the K48‐linked ubiquitination and subsequent degradation of the protein TLE3, which in turn activates the thermogenic gene program in WAT [421].

E3 ligases also control thermogenic activity by regulating the stability of core activators. E3 ligase RNF34 is a specific negative regulator of the metabolic coactivator PGC‐1α, a foundational controller of thermogenesis in brown fat [422]. Functionally, suppressing RNF34 in brown adipocytes increases PGC‐1α protein levels, UCP1 expression, and oxygen consumption [422]. This regulatory axis is physiologically relevant, as stimuli that induce thermogenesis, such as cold exposure, also suppress RNF34 expression, thereby stabilizing PGC‐1α to enhance the metabolic response [422].

The E3 ubiquitin ligase FBXW7 exerts critical, tissue‐specific control over cell fate and lipid metabolism. In adipose tissue, FBXW7 acts as a negative regulator of thermogenesis [448]. It targets the kinase S6K1 for degradation, thereby suppressing brown fat expansion and energy expenditure [448]. As a result, FBXW7 deletion in adipose tissue is metabolically beneficial, protecting mice from diet‐induced obesity and hepatic steatosis by unleashing thermogenic activity [448]. In the liver, FBXW7 is protective, its absence leads to severe steatohepatitis and uncontrolled lipogenesis due to the accumulation of substrates such as Notch [423]. Conversely, during the development of adipose tissue, FBXW7 functions as a key negative regulator, suppressing adipogenesis by targeting the essential transcription factor C/EBPα for proteasomal degradation [424].

E3 ligases also integrate lipid and amino acid metabolism. In diet‐induced obesity, branched‐chain amino acids (BCAAs) promote the E3 ligase Mul1 to mediate the degradation of Akt2, leading to hepatic insulin resistance [449]. The HECT‐type E3 ligase ITCH suppresses adipogenesis by ubiquitinating lipogenic transcription factors such as PPARγ and SREBP‐1c [425]. Beyond lipids, ITCH is essential for maintaining BCAA catabolism, ITCH‐deficient mice develop hepatic steatosis and insulin resistance, while clinical data link ITCH dysfunction in obese individuals to impaired BCAA degradation, elevated circulating BCAAs, and severe hepatic steatosis [425].

Conversely, the SCF complex component FBXW2 negatively regulates BCAA catabolism by targeting key enzymes, such as the BCKDH complex, for degradation [426]. This leads to BCAA accumulation, which can hyperactivate the mTORC1 pathway and induce insulin resistance [426]. In summary, these E3 ligases are key hubs in regulating the complex crosstalk between lipid, amino acid, and insulin signaling pathways in the ubiquitin system, and dysregulation of this network is the core pathogenesis of metabolic diseases.

### 6.3 E3 Ligases in Mitochondrial Dysfunction and Energy Metabolism Regulation

E3 ubiquitin ligases regulate mitochondrial quality control and energy metabolism by regulating the turnover of mitochondrial proteins, maintaining mitochondrial dynamics, and ensuring cellular adaptation to stress. The E3 ligase Parkin serves as the central executor of mitochondrial quality control, primarily by orchestrating the selective removal of damaged mitochondria through a process known as mitophagy [427, 428]. Upon mitochondrial damage, the kinase PINK1 accumulates on the outer mitochondrial membrane (OMM) and initiates a feed‐forward signaling cascade [427, 428]. PINK1 phosphorylates both ubiquitin and Parkin, leading to Parkin's recruitment and full E3 ligase activation on the mitochondrial surface [427, 428]. Activated Parkin then conjugates various polyubiquitin chains (including K6‐, K11‐, and K63‐linked) onto outer membrane proteins, such as VDAC and MFN1/2. These ubiquitin signals serve as scaffolds to recruit autophagy adaptors like p62 and OPTN, which target the damaged mitochondria for lysosomal degradation [427, 428]. Beyond its canonical role in mitophagy, Parkin is a multifaceted E3 ligase with diverse cellular functions. It regulates mitochondrial dynamics, including biogenesis, DNA maintenance, and the formation of mitochondrial‐derived vesicles [450, 451]. Furthermore, Parkin is a key modulator of cellular metabolism, calcium homeostasis, cell death pathways, and various inflammatory responses [452, 453].

In addition to the classic Parkin‐mediated mitophagy pathway, increasing evidence indicates that other E3 ubiquitin ligases also play an indispensable role in mitochondrial quality control [454]. These ligases not only participate in Parkin‐independent mitochondrial clearance pathways, but also exhibit key functions in regulating mitochondrial morphology, dynamics, and protein stability directly related to energy metabolism [454]. MUL1, an OMM E3 ligase, uniquely functions as both a ubiquitin and SUMO ligase to regulate cellular homeostasis [455]. Its ubiquitin ligase activity promotes mitochondrial fission by degrading the fusion protein MFN2, while its SUMO ligase activity stabilizes the fission protein Drp1 [429, 430]. Beyond shaping mitochondrial dynamics, MUL1 targets key regulators such as ULK1, Akt, and p53, directly linking mitochondrial integrity to decisions on mitophagy, cell growth, and apoptosis [456, 457, 458].

MARCH5 (also known as MITOL) is a key E3 ubiquitin ligase on the OMM that governs mitochondrial quality control through multiple mechanisms [431, 459]. It serves as a critical regulator of mitochondrial dynamics, primarily inhibiting excessive fission by targeting components such as Drp1, Fis1, and MiD49 for proteasomal degradation [460, 461, 462]. However, under specific stress conditions, it can also induce fragmentation by degrading fusion proteins such as MFN1 and MFN2 [432, 463]. Beyond shaping the mitochondrial network, MARCH5 directly eliminates misfolded proteins like mSOD1 and PolyQ from the mitochondria to prevent toxic aggregation [464, 465]. It also modulates mitophagy by ubiquitinating the receptor FUNDC1 for degradation and by participating in the initial recruitment of Parkin to damaged mitochondria [433, 466]. Furthermore, MARCH5 regulates inter‐organelle communication by ubiquitinating MFN2 to promote the formation of ER–mitochondria contact sites, which is essential for proper calcium signaling [467].

## 7 Pathological Roles of E3 Ligases in Neurodegenerative Diseases

Neurodegenerative disorders, including AD and PD, represent a formidable challenge to public health [468, 469]. These diseases are defined by the progressive dysfunction and loss of neurons, culminating in severe motor, cognitive, and behavioral impairments [468, 469]. Despite their distinct clinical manifestations, these diseases exhibit common pathological hallmarks at the cellular level [470]. The most significant issue is the fundamental disruption of protein homeostasis, leading to abnormal protein aggregation and the failure of cellular quality control systems meant to prevent it [471, 472].

A central pathological hallmark across many neurodegenerative diseases is the misfolding and aggregation of specific proteins [473]. Under physiological conditions, proteins must adopt precise three‐dimensional structures to function properly. However, stressors such as genetic mutations, oxidative damage, or aging can disrupt this process, causing proteins to misfold and assemble into toxic oligomers and insoluble aggregates [472, 474]. In AD, for instance, the misfolding of amyloid‐β (Aβ) and tau protein leads to the formation of extracellular senile plaques and intracellular neurofibrillary tangles, respectively—lesions that disrupt neuronal integrity and drive cell death [475]. Similarly, in PD, the aggregation of α‐synuclein into Lewy bodies is particularly toxic to the dopaminergic neurons of the substantia nigra [476].

The intimate link between protein degradation and neuropathology was established early, with ubiquitin's discovery in association with brain lesions preceding a full understanding of its broader physiological roles [477]. It is now clear that the UPS is the primary line of defense against the proteotoxic burden imposed by soluble misfolded proteins in neurons [478]. E3 ubiquitin ligases are central to the UPS's specificity, as they recognize aberrant proteins for targeted degradation [9]. Dysfunction of E3 ligases is not merely a consequence of cellular stress but is often a direct driver of pathology [471, 474]. For instance, loss‐of‐function mutations that impair an E3 ligase's ability to clear toxic substrates promote the accumulation of damaged components and subsequent neuronal death [479, 480]. Conversely, the aberrant gain‐of‐function or misregulation of other E3 ligases can lead to the inappropriate destruction of essential neuronal proteins, such as synaptic components or survival factors [481, 482]. Therefore, dysfunction of the E3 ligase can initiate and propagate neurotoxic cascades, thereby leading to a range of neurodegenerative diseases (Table 2) [483, 484, 485].

**TABLE 2:** The pathological roles of E3 ubiquitin ligases in neurodegenerative diseases.

Disease	E3 ligase	Role/function	Mechanism	Reference(s)
Parkinson's disease	Parkin	Pathogenic/protective (loss‐of‐function is pathogenic)	As a key executor of mitophagy, Parkin is recruited by PINK1 to damaged mitochondria. It ubiquitinates outer mitochondrial membrane proteins, marking them for degradation. Loss‐of‐function mutations impair this process, causing dysfunctional mitochondria to accumulate.	[486, 487, 488, 489]
	CHIP	Protective	It mediates the ubiquitination and degradation of LRRK2. Its overexpression reduces LRRK2 levels, while its knockdown is toxic to neurons.	[490, 491]
	Fbxo7 (PARK15)	Protective	It positively regulates mitophagy by helping recruit Parkin to damaged mitochondria. Its deficiency impairs this quality control pathway.	[489]
	Siah‐1	Protective	It directly ubiquitinates and promotes the degradation of α‐synuclein. Its dysregulation may lead to α‐synuclein accumulation and aggregation.	[492]
	NEDD4	Protective	It ubiquitinates α‐synuclein, targeting it to the endolysosomal pathway for degradation and clearance.	[493, 494]
Alzheimer's disease	HRD1	Protective	It mediates ER‐associated degradation (ERAD) of amyloid precursor protein (APP). Reduced HRD1 levels in AD brains lead to APP accumulation and increased Aβ generation.	[495, 496]
	RNF182	Pathogenic	It upregulated in AD brains; contributes to pathology by targeting substrates such as ATP6V0C for degradation.	[497]
	CHIP	Protective	It selectively ubiquitinates hyperphosphorylated tau, marking it for proteasomal degradation and suppressing aggregation. This quality control system is compromised in AD.	[498, 499]
	Parkin	Protective	It reduces Aβ levels and enhances autophagic clearance of Aβ‐induced defects. Its expression and activity are often reduced in AD.	[500]
	NEDD4‐1	Pathogenic	Recruited to synapses by Aβ, where it ubiquitinates and degrades AMPA receptors (AMPARs). This leads to reduced synaptic strength and impaired plasticity.	[501]
	Peli1	Pathogenic	Upregulated in microglia near plaques. It degrades the transcription factor C/EBPβ, which suppresses the expression of the Aβ receptor CD36 and thus impairs microglial Aβ clearance.	[502]
	TRAF6	Pathogenic	It acts as a key adaptor in proinflammatory signaling, particularly the NF‐κB pathway, which is chronically activated in the AD brain and drives neuroinflammation.	[503]
Huntington's disease	Ube3a	Protective	It reduces mutant huntingtin (mHTT) aggregation and suppresses K63‐linked ubiquitination on mHTT; also regulates synaptic function by targeting the protein Arc. Its loss accelerates disease pathology.	[504]
	WWP1	Pathogenic	Upregulated and recruited to mHTT aggregates. It attaches nondegradative K63‐linked ubiquitin chains to mHTT, which inhibits its proteasomal clearance and drives its accumulation.	[505]
	TRAF6	Pathogenic	Upregulated in HD brains. It promotes the formation of larger mHTT aggregates by attaching atypical, nondegradative ubiquitin chains (K6, K27, K29).	[506]
	UBR5	Protective	It drives efficient polyubiquitination and proteasomal degradation of mutant mHTT, suppressing its aggregation in iPSCs.	[507]
	PIAS1 (SUMO E3 ligase)	Pathogenic	It links DNA damage repair (DDR) to HD pathology by SUMOylating the repair enzyme PNKP, reducing PIAS1 restores PNKP activity and improves genomic integrity in HD models.	[508]

### 7.1 Regulatory Mechanisms of E3 Ligases in PD

The pathological hallmarks of PD are the progressive loss of dopaminergic neurons and the accumulation of misfolded α‐synuclein in Lewy bodies [509, 510]. This neurodegeneration is widely recognized as stemming from complex disturbances in cellular homeostasis, particularly involving disruptions in key quality control pathways related to genetic, mitochondrial, and lysosomal mechanisms [511]. Dysfunction of E3 ligases has emerged as a core pathogenic mechanism, with multifaceted roles in mitochondrial quality control, regulation of α‐synuclein aggregation and clearance, and modulation of neuronal survival pathways [512, 513].

One of the most well‐characterized E3 ligases linked to PD is Parkin, and its dysfunction is a primary cause of both familial and sporadic PD [514, 515]. Mutations in PINK1 and Parkin give rise to autosomal recessive, early‐onset PD, with different mutations compromising mitophagy by disrupting distinct stages of the pathway [486, 516]. Under conditions of mitochondrial stress, the kinase PINK1 accumulates on the OMM, where it recruits and activates Parkin [517]. Once activated, Parkin orchestrates the clearance of damaged mitochondria—a process known as mitophagy—by catalyzing the ubiquitination of OMM proteins such as MFN1/2 and VDAC1 [515, 518]. These ubiquitin chains, particularly K27‐ and K63‐linked polymers, serve as a scaffold to recruit autophagy receptors like OPTN and NDP52, thereby flagging the compromised organelle for degradation [515, 518]. Pathogenic loss‐of‐function mutations (e.g., R275W, G430D) or absence of Parkin cripples this essential quality control pathway, leading to the accumulation of dysfunctional mitochondria and conferring a selective vulnerability upon dopaminergic neurons [519]. Mutations in genes beyond PINK1 and Parkin have been shown to impair the PINK1/Parkin‐mediated mitophagy pathway, which is crucial for mitochondrial quality control in PD [512]. Mutations in the LRRK2 gene, particularly the G2019S variant, impair PINK1/Parkin‐mediated mitophagy through enhanced kinase activity, which disrupts mitochondrial clearance and inhibits Parkin binding to its substrates; these deficiencies are reversible upon LRRK2 kinase inhibition [487, 520]. Conversely, the VPS35 D620N mutation, another familial PD‐associated variant, causes reduced mitochondrial membrane potential and disrupts PINK1 stabilization and Parkin translocation to damaged mitochondria, thereby inhibiting mitophagy [521]. Beyond genetic mutations, Parkin's activity is also tightly regulated by PTMs [522]. For instance, the kinase Dyrk1A, which is overexpressed in Down syndrome, negatively regulates Parkin by phosphorylating it at Ser131, thereby inhibiting its mitochondrial translocation and E3 ligase activity [522]. Similarly, S‐nitrosylation, a modification induced by nitric oxide, has been shown to impair Parkin's catalytic function in the brains of PD patients and animal models [488, 523]. These regulatory mechanisms highlight that even a structurally intact Parkin can be rendered inactive, providing novel therapeutic avenues for targeting its upstream regulators to restore mitochondrial homeostasis in neurodegenerative contexts.

As an E3 ligase, CHIP is critically involved in PD pathogenesis, particularly through its regulation of LRRK2. It promotes the degradation of LRRK2, Hsp90 attenuates CHIP‐mediated LRRK2 degradation, an effect that can be blocked by Hsp90 inhibitors [490, 491]. Notably, certain variants such as G2385R, located outside the central ROC–COR–kinase triad, reduce kinase activity and impair interactions with LRRK2 binding partners [524, 525]. The G2385R variant also exhibits lower steady‐state levels and increased protein turnover compared with wild‐type LRRK2 [526]. CHIP directly recognizes multiple regions of LRRK2, including the WD40 domain where G2385R resides, and shows higher affinity for the G2385R mutant than for the wild‐type protein [526]. Overexpression of CHIP reduces levels of both G2385R and WT LRRK2, while CHIP knockdown has the opposite effect and induces neuronal death [526]. These findings suggest CHIP as a promising therapeutic candidate for LRRK2‐linked PD.

Other E3 ligases also contribute to the complex pathology of PD. For example, F‐box only protein 7 (Fbxo7), mutated in some forms of early‐onset Parkinsonism (*PARK15*), is involved in the PINK1/Parkin pathway, acting as a positive regulator of mitophagy [513]. It helps recruit Parkin to damaged mitochondria, and its deficiency impairs this essential quality control process [489, 513]. Similarly, Siah‐1 has been shown to interact directly with α‐synuclein, promoting its ubiquitination and degradation [492]. Dysregulation of Siah‐1 activity could therefore lead to the accumulation and aggregation of α‐synuclein, a cornerstone of PD pathogenesis [527]. The E3 ligase NEDD4 has also been identified as a modulator of α‐synuclein trafficking and clearance, ubiquitinating it to sort it for endolysosomal degradation [493, 494].

In conclusion, E3 ubiquitin ligases are integral to the cellular pathways that are critically compromised in PD [528]. Their functions are essential for maintaining mitochondrial health through mitophagy, ensuring proper protein folding and degradation, and preventing the accumulation of toxic protein aggregates such as α‐synuclein [492]. Therefore, regulating the activity of specific E3 ligases, including enhancing degradation and promoting mitophagy, provides a targeted approach for therapeutic interventions in PD.

### 7.2 Pathological Roles of E3 Ligases in AD

AD, the most common cause of dementia, is characterized by the buildup of Aβ plaques, amyloid precursor protein (APP) and neurofibrillary tangles of tau protein in the brain, leading to progressive cognitive decline and memory loss [529]. Several E3 ligases have been identified that directly regulate the metabolism of APP, the protein from which toxic Aβ peptides are cleaved. A paramount example is HMGCR degradation 1 homolog (HRD1), an E3 ligase embedded in the membrane of the ER that plays a key role in ER‐associated degradation (ERAD) [495, 530]. The ERAD pathway is a quality control mechanism that identifies and targets misfolded or excessive proteins within the ER for retrotranslocation to the cytosol and subsequent proteasomal degradation [531]. Research on AD brains has revealed a significant reduction in HRD1 protein levels, which promotes the degradation of full‐length APP and increases production of Aβ [496]. Consequently, the loss of HRD1 function in AD brains leads to the accumulation of its substrate, APP [496]. This increased substrate pool enhances the amyloidogenic processing of APP by secretase enzymes, resulting in elevated production of Aβ peptides and inducing ER stress, a well‐established feature of AD pathology [532]. The complexity of E3 ligase dysregulation is further illustrated by the brain‐enriched E3 ligase RNF182 [497]. In contrast to HRD1, RNF182 is found to be upregulated in AD brains and may contribute to pathology through its targeting of substrates such as ATP6V0C [497].

The homeostasis of tau protein is also tightly regulated by a dedicated set of E3 ligases, and the failure of this surveillance system is a critical event in the formation of neurofibrillary tangles [498]. CHIP functions as a sophisticated molecular triage system, demonstrating a remarkable ability to discriminate between physiological and pathological forms of tau [499]. It binds to hyperphosphorylated tau with an affinity approximately ten times greater than that for unmodified tau, allowing it to specifically sense and target the disease‐relevant species, which is a critical quality control checkpoint [499]. Upon binding, CHIP catalyzes the ubiquitination of pathological tau, thereby marking it for proteasomal degradation and potently suppressing its propensity to aggregate and form toxic seeds that can propagate pathology [499]. In the context of AD, the function of this crucial triage system is compromised, the overwhelming production of hyperphosphorylated tau may saturate the CHIP system, while the broader collapse of proteostasis or direct inhibitory effects of Aβ may impair CHIP's activity, allowing pathological tau to escape degradation and proceed down the aggregation cascade [499]. Another important E3 ligase implicated in tau pathology is Parkin, which is most famously associated with familial forms of PD but also plays a role in AD [533]. Parkin can reduce Aβ levels, enhance autophagic clearance of Aβ‐induced defects, and aggravation of the phosphorylation of Tau protein in AD, and its expression and activity are often reduced in AD [500, 533].

The role of E3 ubiquitin ligases in AD extends far beyond protein degradation, such as synaptic dysfunction and neuroinflammation [501]. Synaptic failure is one of the main biological correlates of cognitive decline in AD, and E3 ligases are key players in remodeling the synaptic proteome [534]. Aβ has been shown to trigger the recruitment of the HECT E3 ligase NEDD4‐1 to dendritic spines [501]. Once NEDD4‐1 localizes to the synapse, it can perform its normal function of ubiquitinating and degrading AMPA receptors (AMPARs) [501]. The direct consequences are a reduction in synaptic strength, a physical loss of dendritic spines, and an impairment of synaptic plasticity, providing a precise molecular mechanism by which Aβ drives cognitive deficits [501].

Other E3 ligases act as intrinsic regulators of synaptic function and are dysregulated in AD. The brain‐enriched E3 ligase PRAJA1 functions as a homeostatic “molecular brake” on memory formation; its levels are normally downregulated during long‐term potentiation to permit synaptic strengthening [535]. In AD models, PRAJA1 is dysregulated, contributing to synaptic deficits through its targeting of key structural proteins like spinophilin [535].

Chronic neuroinflammation, mediated primarily by the brain's resident immune cells, microglia and astrocytes, is a major driver of neurodegeneration in AD [536]. The E3 ligase Peli1 is significantly upregulated in microglia surrounding plaques in both AD mouse models and human patient brains [502]. Peli1 targets the transcription factor C/EBPβ for ubiquitination and degradation, C/EBPβ is essential for the expression of the scavenger receptor CD36, a key receptor that microglia use to recognize and phagocytose Aβ [502]. By promoting the degradation of C/EBPβ, the elevated Peli1 levels effectively shut down the microglial Aβ clearance pathway, thus exacerbating plaque deposition and sustaining the inflammatory stimulus [502].

TRAF6 is a pivotal adaptor protein in multiple proinflammatory signaling cascades, most notably the NF‐κB pathway, which is chronically activated in the AD brain and drives the expression of inflammatory cytokines. IL‐17 and gastrodin can reduce neuroinflammation and microglial activation in AD models through the TRAF6/NF‐κB pathway [503, 537]. In addition, in AD models, the key protein TRIM9 reduces neuroinflammation by inhibiting the NF‐κB signaling pathway, thereby delaying the decline of cognitive function [538].

### 7.3 Pathological Roles of E3 Ligases in Huntington's Disease

HD is an autosomal dominant, fatal neurodegenerative disorder precipitated by a CAG trinucleotide repeat expansion within the huntingtin (HTT) gene [539, 540]. This gene is located on chromosome 4, and the CAG expansion in exon 1 produces a mutant huntingtin protein (mHTT) that is abnormally modified posttranslationally, leading to transcriptional, immune, and mitochondrial dysfunction; it also serves as the earliest detectable biomarker in the serum of HD patients [541, 542]. The HECT domain E3 ligase Ube3a is a critical neuroprotective factor in HD. Overexpression of Ube3a has been shown to reduce mHTT aggregation and concurrently suppress the accumulation of aggregation‐prone K63‐linked ubiquitin chains on the mutant protein [543]. In addition, the E3 ubiquitin ligase Ube3a plays a crucial neuroprotective role in HD, as its selective removal from the brains of HD mice accelerates the disease phenotype and mortality [504]. This exacerbated pathology is associated with a significant increase in less‐ubiquitinated mutant huntingtin aggregates and a reduction of the key striatal marker DARPP‐32 [504]. Ube3a is essential for normal synaptic function and plasticity, partly through its regulation of the synaptic protein Arc [544]. The loss of functional Ube3a in HD leads to an accumulation of synaptic Arc and a reduction in AMPA receptor levels, providing a direct molecular link between mHTT aggregation, E3 ligase dysfunction, and the early cognitive deficits characteristic of the disease [544].

In contrast to neuroprotective enzymes, the HECT domain E3 ligase WWP1 functions as a key pathogenic factor in HD [505]. Upregulated in HD models and recruited to mHTT aggregates, WWP1 decorates mHTT with K63‐linked ubiquitin chains [505]. This nondegradative signal actively inhibits proteasomal clearance, driving mHTT accumulation and cytotoxicity and thus establishing WWP1 as a validated therapeutic target [505]. Moreover, the RING E3 ligase TRAF6 contributes to HD pathology through a distinct, proaggregation mechanism. TRAF6 is upregulated in the brains of HD patients and is recruited to mHTT inclusions [506]. It promotes the formation of larger aggregates by attaching atypical, nondegradative ubiquitin chains via Lys6, Lys27, and Lys29 linkages [506]. This action highlights how specific E3 ligases can corrupt the ubiquitin code in noncanonical ways to directly drive protein aggregation [506].

The greater vulnerability of neurons to misfolded proteins is driven by the cell‐specific inhibition of the E3 ligase CHIP [545]. Unlike astrocytes, neurons express high levels of the inhibitor HspBP1, which suppresses CHIP‐mediated protein clearance [545]. Knocking down HspBP1 in neurons unleashes CHIP's protective activity, reducing mutant huntingtin aggregation and ameliorating neuropathology in HD models [545]. The inherent ability of induced pluripotent stem cells (iPSCs) to suppress mutant mHTT aggregation is critically dependent on the E3 ligase UBR5 [507]. High endogenous expression of UBR5 in iPSCs drives the efficient polyubiquitination and proteasomal degradation of mHTT [507]. This protective mechanism is confirmed by findings that UBR5 knockdown induces aggregation in HD‐iPSCs, while its overexpression reduces aggregates in various HD models [507].

Recent studies have identified the SUMO E3 ligase PIAS1 as a critical mediator linking DNA damage repair (DDR) to HD pathology. As a component of the transcription‐coupled repair complex, PIAS1 SUMOylates the repair enzyme PNKP [508]. Reducing PIAS1 in both HD mouse models and patient‐derived iPSC neurons normalized disease‐associated transcriptional dysregulation, restored PNKP enzymatic activity impaired by mutant HTT, and improved genomic integrity [508].

## 8 Therapeutic Strategies Involving E3 Ligase Modulation

The UPS represents a critical PTM in the regulation of cellular homeostasis. Its role in the targeted degradation of proteins has been intricately correlated to the pathogenesis of diverse cancers, underscoring its importance in maintaining cellular integrity [546]. E3 ligase, a key component of UPS, identifies and binds ubiquitin to target substrates, thereby labeling these proteins for degradation. The malfunction of E3 ligases has been demonstrated to affect a number of key biological processes, including gene expression, cell cycle, signal transduction and DDR [547].

### 8.1 E3 Ligase‐Targeted Therapy

Since the first proteasome inhibitor, Bortezomib, was approved by the United States Food and Drug Administration for the treatment of mantle cell lymphoma and multiple myeloma, scientists have conducted a series of studies to explore the potential of UPS as a strategy for cancer therapy [67, 548]. As the most important component of the UPS, E3 ligase has the highest substrate specificity with better efficacy and lower side effects [549]. In the treatment of cancer, novel therapeutics targeting E3 ligases are currently being developed with the objective of modulating protein homeostasis and enhancing chemosensitivity, presenting a potential avenue for overcoming drug resistance and improving clinical outcomes.

A primary example is the E3 ligase MDM2, which contains a C‐terminal RING‐finger domain that facilitates the ubiquitination of the p53 tumor suppressor, thereby targeting it for proteasomal degradation [550]. This interaction occurs when key hydrophobic residues of p53, namely Phe19, Trp23, and Leu26, dock into a hydrophobic binding pocket on the N‐terminal domain of MDM2 [551]. Consequently, a major therapeutic approach involves the development of small molecules that competitively bind to this MDM2 pocket, mimicking the essential p53 residues and disrupting the p53–MDM2 complex, which prevents p53 degradation and restores its tumor‐suppressive activities [552, 553]. A number of small molecules have been developed to inhibit the E3 ligase activity of MDM2 through direct binding. The Nutlin family of *cis*‐imidazoline compounds was among the first small molecules designed to specifically inhibit the p53–MDM2 interaction by mimicking the binding of Phe19, Trp23, and Leu26 [554, 555]. This class has since expanded to include next‐generation inhibitors with improved potency and clinical potential, such as Idasanutlin (RG7388) and SAR405838 [556, 557]. Beyond direct competitive inhibition, an alternative strategy to block E3 ligase function is to induce PTMs of the substrate that abrogate ligase binding. The phosphorylation of p53 at residues Ser15, Thr18 and Ser20 has been shown to prevent its interaction with MDM2, thereby sparing p53 from ubiquitination and degradation [558, 559]. Certain natural products, including indole‐3‐carbinol and isokotomolide A, have been identified to act through this mechanism, promoting the phosphorylation of p53 at Ser15 and subsequently stabilizing the protein [560, 561].

In summary, targeting E3 ubiquitin ligases has emerged as a highly promising drug development strategy [562]. By modulating the interactions between E3 ligases and their substrates, intracellular protein homeostasis can be restored and pathogenic proteins cleared, thereby inhibiting carcinogenic or disease‐causing signaling pathways [563].

### 8.2 PROTAC

**FIGURE 6:**
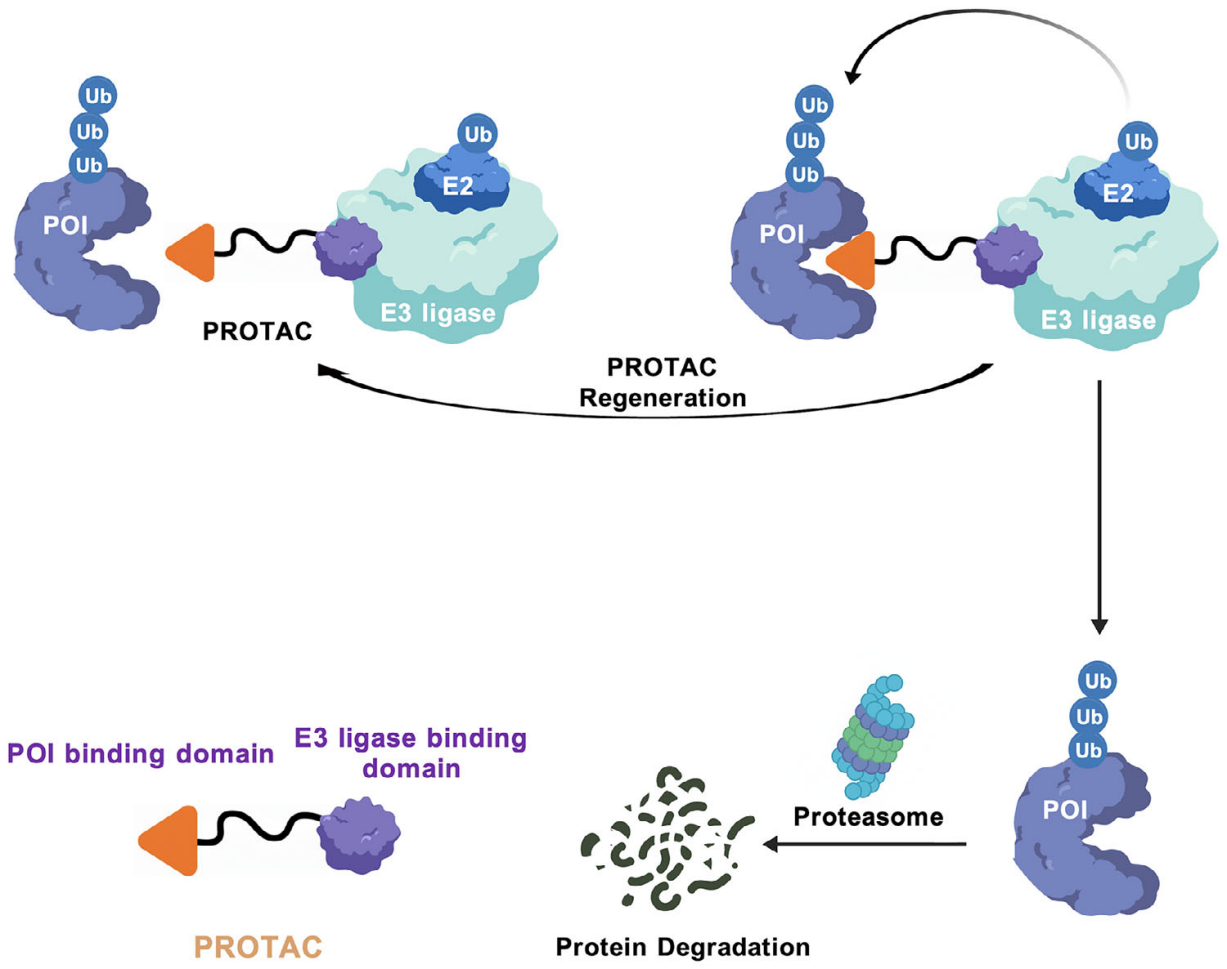
Mechanism of action of proteolysis‐targeting chimeras (PROTACs). PROTACs represent an innovative therapeutic strategy that co‐opts the cell's endogenous UPS to selectively eliminate pathogenic proteins. A PROTAC is a heterobifunctional molecule composed of three key parts: a ligand that binds the protein of interest (POI), a second ligand that recruits an E3 ubiquitin ligase, and a chemical linker that connects them. By simultaneously engaging both the POI and the E3 ligase, the PROTAC induces the formation of a ternary POI–PROTAC–E3 complex. This forced proximity enables the E3 ligase to efficiently catalyze the transfer of ubiquitin (Ub) from a ubiquitin‐charged E2 enzyme to the surface of the POI, resulting in its polyubiquitination. The polyubiquitinated POI is then recognized by the 26S proteasome and targeted for degradation. After the POI is destroyed, the PROTAC molecule is released and can engage in further catalytic cycles of degradation, offering a powerful alternative to traditional occupancy‐based inhibitors.

In addition, PROTACs, an emerging anticancer therapeutic approach closely associated with E3 ligases, have been demonstrated to exhibit superior efficacy, selectivity, and ability to overcome drug resistance in comparison with traditional protein inhibitors (Figure 6). PROTAC molecules are heterobifunctional in nature, typically comprising three essential elements: a ligand that binds to the protein of interest (POI), a linker, and another ligand that recruits an E3 ubiquitin ligase [564, 565]. Upon simultaneously engaging both the target protein and the E3 ligase, PROTACs promote the formation of a ternary POI–PROTAC–E3 complex [566, 567]. This proximity‐driven interaction hijacks the UPS, resulting in polyubiquitination of the POI and its subsequent proteasomal degradation [568]. In essence, PROTACs hijack the cell's natural protein degradation machinery, mimicking the E3 ligase's substrate recognition to efficiently and specifically eliminate the target protein. PROTACs have been developed as a new technology for TPD, offering a promising alternative to conventional targeted gene strategies. PROTACs facilitate the intrinsic protein degradation mechanism, enabling the selective degradation of the POI or target protein. PROTACs offer several advantages in terms of adverse effects, drug resistance and off‐target effects [569].

The development of nonpeptidic ligands for cereblon (CRBN) and von Hippel–Lindau (VHL) has been particularly transformative, as these ligands support the design of PROTACs with drug‐like features and have propelled their entry into clinical trials, especially in oncology [228, 570, 571]. Within this context, bromodomain and extraterminal (BET) proteins stand out as compelling targets because of their role in histone recognition, transcriptional regulation, and oncogene expression [572, 573]. BET‐directed PROTACs employing CRBN or VHL successfully degrade these regulators [574]. Nonetheless, incomplete tumor eradication has been observed, largely attributed to resistance mutations that destabilize the ternary complex [574, 575]. Since productive ubiquitination requires sufficiently stable ligase–substrate interactions, the search for alternative E3 ligases beyond CRBN and VHL has become a critical direction [567].

A related challenge is the accurate identification of native E3 ligase substrates. Substrate mapping is complicated by transient and redundant interactions, as well as the rapid turnover of ubiquitinated proteins. Classical methods including yeast two‐hybrid assays, protein microarrays, global protein stability profiling, and proteomics have provided useful insights but remain limited [576]. To overcome these constraints, newer approaches such as BioE3, which applies BirA–E3 fusions for site‐specific biotinylation, substrate‐trapping strategies using TUBE and antiubiquitin remnant antibodies, and the E‐STUB proximity labeling platform, have emerged as powerful tools to improve substrate discovery and thereby facilitate rational PROTAC development [577, 578, 579]. Moreover, the in silico design of PROTACs has been significantly advanced by a variety of computational methods, ranging from non‐AI approaches focused on accurately modeling the ternary complex to the application of AI‐based generative models for the de novo design of linkers and entire PROTAC molecules [580]. These tools address key challenges throughout the design pipeline, including target tractability assessment, E3 ligase selection, and prediction of the final molecule's degradation efficacy.

### 8.3 Clinical Trials Targeting E3 Ligases

In recent years, the clinical translation of E3 ubiquitin ligases has accelerated due to the development of therapies based on E3 ubiquitin ligases, such as PROTACs [581]. An increasing number of E3–centric drug combinations are undergoing clinical trials for cancer, autoimmune diseases, and other conditions. Based on the Clinicaltrials.gov database, we systematically retrieved and summarized a series of trials in various fields including oncology and immunology that utilize E3 ubiquitin ligases in their mechanisms, such as PROTACs and direct E3 modulators (Table 3).

**TABLE 3:** Clinical studies targeting E3 ubiquitin ligases.

NCT	Disease	Drug	E3 ligase involved	Mechanism	Phase	Key objectives/findings
NCT06767956	Non‐Hodgkin lymphoma (NHL)	Golcadomide (CC‐99282), nivolumab	Cereblon	Golcadomide: A cereblon E3 ligase modulator drug (CELMoD) agent that binds to the cereblon E3 ligase, inducing the degradation of target proteins. This results in immunomodulatory and direct tumor‐killing activities.	Phase 1/phase 2	Phase 1: determine the maximum tolerated dose and recommended phase 2 dose of golcadomide with standard‐dose nivolumab. Phase 2: evaluate the objective response rate per lugano criteria, targeting an ORR of ≥45%.
NCT06209619	NHL	Golcadomide (CC‐99282), rituximab	Cereblon	Golcadomide is a CELMoD that binds to the cereblon E3 ligase, inducing the targeted degradation of transcription factors.	Phase 1	To determine the safety profile and maximum tolerated dose of the golcadomide and rituximab combination. The study is listed as recruiting.
NCT06834373	NHL	Golcadomide, rituximab	Cereblon	Golcadomide is a CELMoD that binds to the cereblon E3 ligase, inducing the targeted degradation of transcription factors.	Phase 2	To evaluate the disease control rate after two cycles of combination therapy. To assess safety, tolerability, and the proportion of patients who can successfully proceed to CAR‐T therapy (bridging efficacy).
NCT05177536	Multiple myeloma	Iberdomide	Cereblon	It acts as a potent cereblon E3 ligase modulator.	Phase 2	Determine the feasibility, safety, and efficacy of iberdomide maintenance post‐ASCT; assess the proportion completing 1 year of therapy, median PFS, MRD‐negativity rates, OS, and safety.
NCT04855136	Multiple myeloma	bb2121 (Ide‐cel), CC‐220 (iberdomide)	Cereblon	CC‐220 is a cereblon E3 ligase modulator agent. It binds to the cereblon E3 ligase complex, altering its substrate specificity to induce the ubiquitination and subsequent proteasomal degradation of target proteins.	Phase 1/phase 2	Phase 1: determine dose limiting toxicity rates. Phase 2: evaluate the complete response rate. The study is listed as terminated.
NCT06896916	Multiple myeloma	Etentamig, iberdomide	Cereblon	Iberdomide is a CELMoD agent that binds to the CRBN E3 ligase.	Phase 1/phase 2	To assess the safety, tolerability, and dose‐limiting toxicities of etentamig in combination with iberdomide to determine a tolerable dose for phase 1.
NCT06048250	Multiple myeloma	Mezigdomide (CC‐92480)	Cereblon	It binds to the cereblon protein, which triggers the degradation of target proteins. This leads to myeloma cell death and may extend the persistence of CAR T‐cells.	Phase1	To evaluate the safety, tolerability, and optimal dose of mezigdomide following idecabtagene vicleucel. The study is currently recruiting.
NCT06518551	Multiple myeloma	Iberdomide, elotuzumab, dexamethasone	Cereblon	Iberdomide is a CELMoD that binds to cereblon, inducing the degradation of target proteins	Phase 1/phase 2	Phase 1: determine the maximum tolerated dose and dose limiting toxicities of the combination. Phase 2: evaluate the 12‐month progression‐free survival rate. The trial is currently recruiting.
NCT05434689	Multiple myeloma	Iberdomide, daratumumab, dexamethasone, carfilzomib	Cereblon	Iberdomide is a CELMoD that binds to cereblon, inducing the degradation of target proteins.	Phase 1/phase 2	Phase 1: determine the maximum tolerated dose and dose limiting toxicities of the combination. Phase 2: evaluate the 12‐month progression‐free survival rate. The trial is currently recruiting.
NCT06050512	Multiple myeloma	Mezigdomide (CC‐92480), ixazomib, dexamethasone	Cereblon	Mezigdomide is a CELMoD agent that binds to the cereblon E3 ligase, promoting the ubiquitination and maximal degradation of transcription factors.	Phase 1/phase 2	Phase 1: determine the recommended phase 2 dose by assessing dose‐limiting toxicities. Phase 2: evaluate the overall response rate of the combination therapy per IMWG criteria. The trial is currently withdrawn.
NCT05981209	Multiple myeloma	Mezigdomide (CC‐92480), elotuzumab, dexamethasone	Cereblon	Mezigdomide is a CELMoD agent that binds to the cereblon E3 ligase, promoting the ubiquitination and maximal degradation of transcription factors.	Phase 1b	Evaluate the safety, tolerability, and determine the recommended phase 2 dose of mezigdomide (CC‐92480) combined with elotuzumab and dexamethasone. The trial is currently recruiting.
NCT07172126	Prostate cancer	TQB3201	Cereblon	TQB3201 is an oral proteolysis‐targeting chimera (PROTAC) that links the androgen receptor (AR) to the cereblon E3 ligase, inducing the targeted degradation of wild‐type and mutant AR.	Phase 1/phase 2	Phase 1: evaluate the safety, tolerability, pharmacokinetics, maximum tolerated dose, and recommended phase 2 dose. Phase 2: assess preliminary efficacy, including radiographic progression‐free survival. The trial is listed as “not yet recruiting.”
NCT07104110	Prostate cancer	QLH12016	PROTAC (E3 ligase recruiter), acts as androgen receptor degrader	QLH12016 is an oral PROTAC (proteolysis‐targeting chimera) designed to induce the degradation of the androgen receptor (AR).	Phase 1/phase 2	Phase 1b: to evaluate the safety, tolerability, dose‐limiting toxicity, and determine the recommended phase 2 dose. Phase 2: to assess preliminary antitumor activity, primarily objective response rate and PSA response. The trial is listed as “not yet recruiting.”
NCT07198633	Prostate cancer	QLH12016; QLC5508; abiraterone acetate; enzalutamide	PROTAC (recruited by QLH12016)	QLH12016: an oral PROTAC designed to recruit an E3 ligase, leading to the ubiquitination and degradation of the androgen receptor (AR).	Phase 1b/phase 2	Phase 1: determine the recommended phase 2 dose and assess safety and tolerability. Phase 2: evaluate preliminary antitumor activity, including PSA50 response and objective response rate.
NCT06620302	Relapsed or refractory solid tumors and fibrolamellar carcinoma	DT2216, irinotecan	PROTAC, von Hippel–Lindau (VHL)	DT2216, a PROTAC targeting Bcl‐xL for degradation via VHL E3 ligase	Phase 1/phase 2	Phase 1: To estimate the maximum tolerated dose and recommended phase 2 dose of DT2216 combined with irinotecan. Phase 2: to preliminarily define the antitumor activity in solid tumors and in a specific cohort for FLC.
NCT02848001	Relapsed or refractory acute myeloid leukemia or relapsed or refractory higher‐risk myelodysplastic syndromes	CC‐90009	Cereblon	CC‐90009 is a novel CELMoD designed to bind to and modulate the cereblon E3 ligase complex.	Phase 1	The primary objective was to evaluate the safety, tolerability, and determine the maximum tolerated dose and recommended phase 2 dose of CC‐90009. The trial was terminated due to a lack of efficacy in the short‐term acute phase.
NCT04606446	Advanced or metastatic solid tumors (including ER+ HER2− breast cancer, CRPC, and NSCLC)	PF‐07248144, PF‐07850327, ARV‐471, vepdegestrant	PROTAC, cereblon	Vepdegestrant, a PROTAC degrader that recruits the cereblon E3 ligase to the estrogen receptor (ER), inducing ER degradation.	Phase 1/phase 2A	Evaluate the safety, tolerability, and pharmacokinetics; determine the maximum tolerated dose and recommended dose for expansion of PF‐07248144 as a single agent and in combinations. Assess preliminary antitumor activity. Findings: PF‐07248144 monotherapy was well tolerated, showed a manageable safety profile, and demonstrated preliminary antitumor activity in ER+ HER2− metastatic breast cancer.
NCT06206837	Breast cancer	Vepdegestrant, PF‐07220060	PROTAC, cereblon	Vepdegestrant, a PROTAC degrader that recruits the cereblon E3 ligase to the ER, inducing ER degradation.	Phase 1b/2	Phase 1b: evaluate the safety, tolerability, and recommended phase 2 dose of the combination. Phase 2: assess the preliminary antitumor activity. The study is active but not currently recruiting.
NCT05654623	Breast cancer	Vepdegestrant (ARV‐471)	PROTAC, cereblon	Vepdegestrant, a PROTAC degrader that recruits the cereblon E3 ligase to the ER, inducing ER degradation.	Phase 3	To compare progression‐free survival of vepdegestrant versus fulvestrant in patients who progressed after prior endocrine therapy and a CDK4/6 inhibitor.
NCT05548127	Breast cancer	Vepdegestrant (ARV‐471), abemaciclib	PROTAC, cereblon	Vepdegestrant, a PROTAC degrader that recruits the cereblon E3 ligase to the ER, inducing ER degradation.	Phase 1b/2	Phase 1b: determine the recommended phase 2 dose and assess dose‐limiting toxicities of the combination. Phase 2: evaluate preliminary antitumor activity, defined by objective response, and safety of the combination at the RP2D.
NCT05501769	Breast cancer	Vepdegestrant (ARV‐471), everolimus	PROTAC, cereblon	Vepdegestrant, a PROTAC degrader that recruits the cereblon E3 ligase to the ER, inducing ER degradation.	Phase 1b	To assess the safety, tolerability, and determine the recommended phase 2 dose of the combination therapy.
NCT06964009	Ovarian cancer	DT2216, paclitaxel	PROTAC, VHL	DT2216, a PROTAC targeting Bcl‐xL for degradation via VHL E3 ligase	Phase 1	To determine the maximum tolerated dose, recommended phase 2 dose, and safety of DT2216 combined with paclitaxel. Secondary objectives include evaluating overall response rate and progression‐free survival.
NCT06586957	Advanced/metastatic solid tumors	NKT3964	PROTAC	NKT3964 is an oral PROTAC that forms a ternary complex by simultaneously binding CDK2 and an E3 ubiquitin ligase. This proximity induces the ubiquitination and subsequent proteasomal degradation of CDK2. Depletion of the CDK2 protein prevents the phosphorylation of retinoblastoma (Rb), blocking the G1/S cell cycle transition.	Phase 1	Evaluate safety, tolerability, pharmacokinetics, and preliminary antitumor activity; determine the recommended dose for expansion and recommended phase 2 dose.
NCT03161483	Systemic lupus erythematosus (SLE)	CC‐220 (iberdomide)	Cereblon	Iberdomide's clinical efficacy in SLE correlated with a reduction in the type I IFN gene signature. The drug selectively rebalanced immune abnormalities by suppressing type I IFN and B cell pathways while elevating IL‐2 and Tregs.	Phase 2	Objective: To evaluate the efficacy and safety of three doses of CC‐220 (0.45, 0.3, or 0.15 mg) compared with placebo in adults with active SLE. Proportion of subjects achieving an SLE Responder Index 4 (SRI‐4) response at week 24. Findings: The 0.45 mg dose met the primary endpoint, demonstrating a significantly higher SRI‐4 response rate compared with placebo.

## 9 Conclusion and Perspective

E3 ubiquitin ligases play a crucial role in cellular homeostasis, and their dysfunction is a key pathogenic mechanism in numerous human diseases. In carcinogenesis, this is manifested by the functional dichotomy of E3 ligases acting as oncogenes that degrade tumor suppressors or as tumor suppressors that eliminate oncoproteins. In neurodegenerative disorders, the failure of specific E3 ligases to clear toxic protein aggregates is a direct driver of pathology, leading to the accumulation of species like α‐synuclein and tau. In autoimmune and inflammatory diseases, dysregulation of E3 ligase disrupts the regulation of immune signaling, leading to the breakdown of self‐tolerance and chronic inflammation. Similarly, in cardiovascular and metabolic syndromes, aberrant E3 ligase activity drives pathological remodeling, compromises mitochondrial quality control, and disrupts the homeostatic control of glucose and lipid metabolism.

Consequently, the properties that establish E3 ligases as drivers of disease also position them as compelling targets for therapeutic intervention. Their enzymatic nature and inherent substrate specificity offer a powerful handle for pharmacological intervention, a potential now being realized through diverse and innovative strategies. The development of small‐molecule inhibitors, such as the Nutlin family and AMG‐232, targets E3 ubiquitin ligases to regulate downstream substrates and restore cellular homeostasis. Beyond direct inhibition, the therapeutic landscape is being revolutionized by the advent of TPD technologies, most notably PROTACs. This paradigm‐shifting approach repurposes the cell's own degradation machinery, hijacking specific E3 ligases to induce the selective elimination of previously “undruggable” pathogenic proteins. The success of PROTACs that recruit common E3 ligases such as CRBN and VHL has catalyzed a new wave of drug discovery, offering advantages in efficacy, selectivity, and the potential to overcome conventional drug resistance across multiple disease contexts.

Despite this therapeutic promise, several significant challenges must be addressed to fully realize the potential of targeting E3 ligases. The vast majority of the over 600 E3 ligases in the human genome remain therapeutically unexploited, largely due to a limited understanding of their substrates and a lack of specific ligands. Expanding the pool of recruitable E3 ligases is crucial for developing tissue‐specific degraders and overcoming resistance mechanisms caused by mutations in core UPS components. However, no universal approach exists for developing E3 ligases, as these molecules present chemical, structural, or safety challenges. The emergence of innovative proteomic and proximity‐labeling techniques, such as BioE3, coupled with advanced computational and AI‐driven models for rational PROTAC design, offers a promising path forward to illuminate these complex networks.

In summary, the UPS, particularly E3 ubiquitin ligases, has been established as critical regulators of cellular homeostasis. This review has detailed the role of E3 ligases in various human diseases, including cancer, neurodegenerative diseases, cardiovascular diseases, autoimmune and inflammatory diseases, and metabolic syndrome. E3 ligases play a central role in determining the fate of critical proteins, making them highly attractive therapeutic targets. Developing therapies to modulate their activity represents an important and rapidly evolving frontier, encompassing strategies ranging from small‐molecule inhibitors that block dysfunctional E3 ligases to emerging TPD technologies such as PROTACs. This review provides an in‐depth overview of the critical roles of E3 ligases in diverse diseases and E3 ligase‐based therapeutic approaches, aiming to lay the groundwork for novel treatments and targeted therapeutics. Continued in‐depth research on E3 ligases and the development of targeted therapeutics will advance E3 ligase‐targeted therapies, ultimately delivering significant clinical benefits to patients.

## Author Contributions

H.W. drafted the manuscript and prepared figures and tables. H.L. and J.P. drafted the manuscript. Y.L. and J.G. edited and revised the manuscript. Q.Q. and X.H. conceived and revised the manuscript for final submission. All authors have read and approved the final manuscript.

## Ethics Statement

The authors have nothing to report.

## Conflicts of Interest

The authors declare no conflicts of interest.

## Data Availability

The authors have nothing to report.
